# Supplementary material for: Multiple yet switchable hydrogen-bonded organic frameworks with white-light emission
Source: Nat Commun. 2022 Apr 6;13:1882. doi: 10.1038/s41467-022-29565-1 (PMC8987099; doi:10.1038/s41467-022-29565-1)
Supplement: Supplementary file 1 — Supplementary Information [file 41467_2022_29565_MOESM1_ESM.pdf]

Supplementary Information

Multiple yet switchable hydrogen-bonded organic frameworks with white-light emission

Shi *et al.*

Supplementary Methods

All reagents and 4CN were commercially available and used after further distally purification. NMR spectra were recorded on a Bruker Advance DMX 400 MHz spectrophotometer in deuterated solvents and referenced using residual solvent peaks or by using tetramethylsilane (TMS) as the internal reference. Chemical shifts were reported relative to residual solvent signals. Absorption and photoluminescence spectra were recorded on a UV-vis Spectrophotometer U-3900 spectrophotometer and a FS 5 spectrofluorometer, repectively. The lifetimes of fluorescence were acquired with a NanoLED laser with the excitation peak at 374 nm or a Horiba Fluoro max plus spectrophotometer equipped with a xenon arc lamp (Xe900). Lifetime data was analyzed with DataStation v6.6 (Horiba Scientific). Absolute fluorescence quantum yields were measured on a Hamamatsu Quantaurus QY spectrometer or a Fluormax-4p spectrometer. Time-resolved fluorescence spectra were measured on a time-correlated single-photon-counting fluorescence spectrometer (Edinburgh Instruments FLS920) with laser (343 nm) used as excitation sources. Fluorescent microscopy images were acquired at an excitation wavelength of 365 nm using a Nikon Ni-U Fluorescence Microscope. The DFT and TD-DFT calculations were performed on a Gaussian 09 program. Thermogravimetric analysis (TGA) was carried out on a Shimadzu-50 at a heating rate of 10 °C/min under dry nitrogen. Differential scanning calorimetry (DSC) were performed with a DSC Q2000 analyzer under nitrogen atmosphere with a heating rate of 5 °C/min. The power X-ray diffraction (PXRD) patterns of all samples were collected by a X-ray diffractometer (Japan Rigaku D/MAX-γA) with Cu-Kα radiation (λ = 0.154 nm). Single crystal data sets were collected on a Stoe Stadivari instrument. The high pressure adsorption N_2 isotherm were collected using a ASAP2460 (Micromeritics Instruments) volumetric analyzer. The sample was activated first at 110 °C for 12 hours, Then, the Brunauer-Emmett-Teller (BET) surface areas were calculated from the adsorption data in the relative pressure (P/P_0) ranging from 0 to 0.1.

Supplementary Figures and Tables

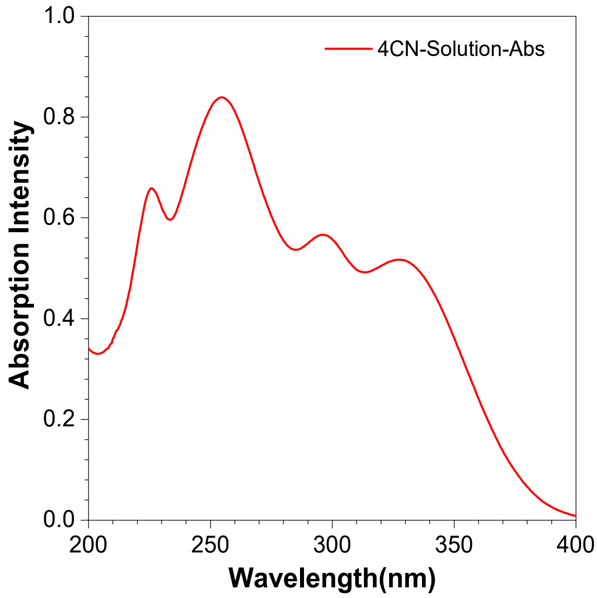

Supplementary Figure 1. UV-Vis spectra of 4CN (25 μM) in THF solution.

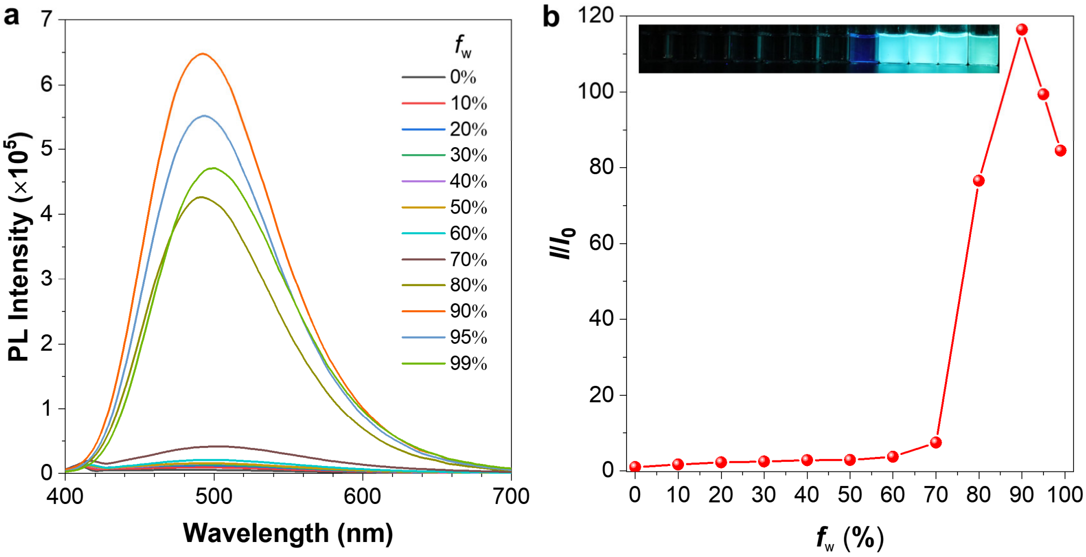

Supplementary Figure 2. a PL spectra of 4CN (10.0 μM) in acetone/H_2O mixtures with different H_2O fractions. **b** Changes of relative PL intensity of 4CN in acetone/H_2O mixtures with different f_w based on the intensity change at $\lambda = 495$ nm. $\lambda_{ex} = 365$ nm. f_w indicates H_2O fraction. I: PL intensity at different f_w. I_0: PL intensity at $f_w = 0\%$. Insert: the photographs of 4CN taken under UV illumination with different H_2O fractions.

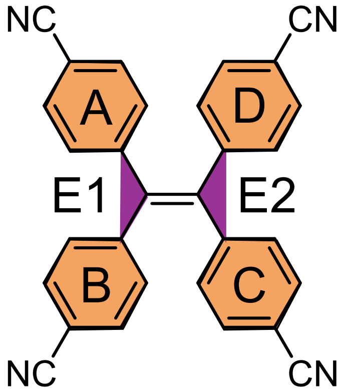

Supplementary Figure 3. Illustration used to define the planes of the ethylene core and phenyl rings in the 4CN molecule. The four phenyl rings in TPE moieties are defined as planes A, B, C and D. Planes E1 and E2 used to define the planes of the ethylene core, respectively. In terms of the effect on fluorescence emission, dihedral angles (*θ*) between the phenyl rings (A, B, C, D) and the plane of the ethylene core (E1, E2) plays an important role in 4CN.

The ground state geometries were optimized using the density function theory (DFT) method with B3LYP hybrid functional at the basis set level of 6-31G(d,p) in gas phase. The combined quantum mechanics and molecular mechanics (QM/MM) method with two-layer ONIOM approach was used to simulate the properties in the solid state and the solid-phase computational model was built based on the X-ray crystal structure. The central molecule acted as the high layer (QM) at the B3LYP/6-31G(d,p) level, and the surrounding molecules were treated as the low layer (MM) using Universal Force Field (UFF). Beside, molecules of MM part are frozen during the QM/MM geometry optimizations for ground state. All these calculations were carried out in the Gaussian 16 package.

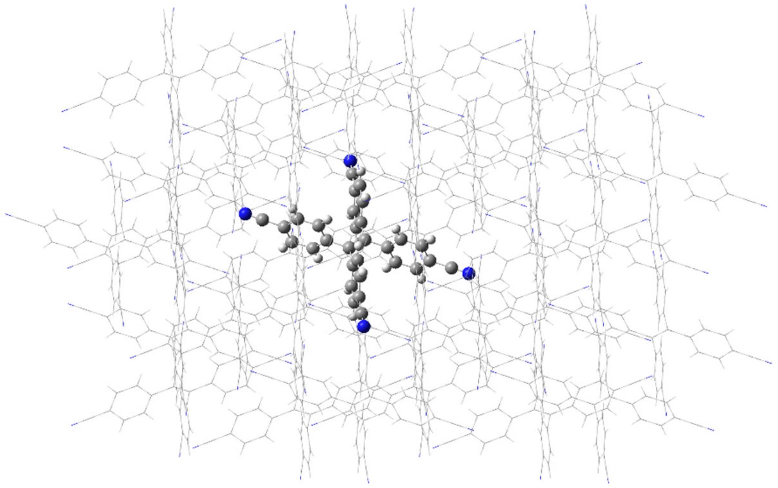

Supplementary Figure 4. ONIOM model: surrounding molecules are regarded as the low layer and the centered 4CN is treated as the high layer.

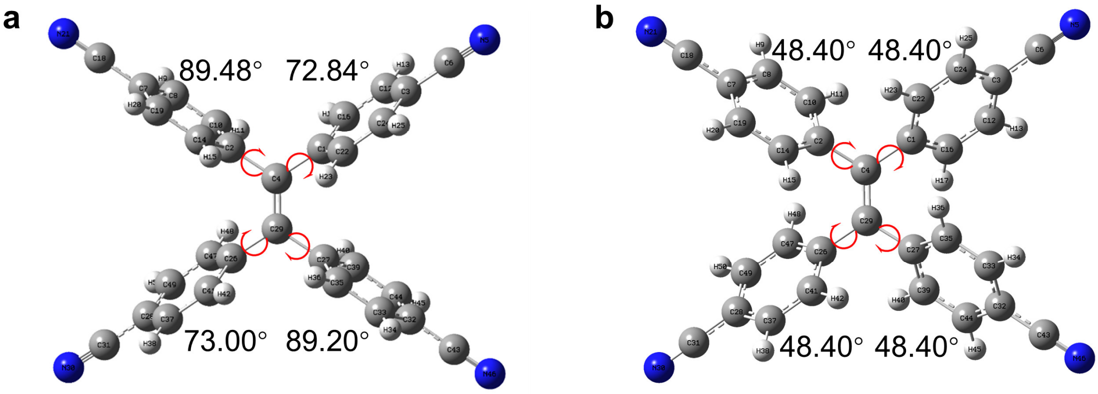

Supplementary Figure 5. Calculated dihedral angle distribution of 4CN(Non1) **a** in a solid environment and **b** in a gaseous environment.

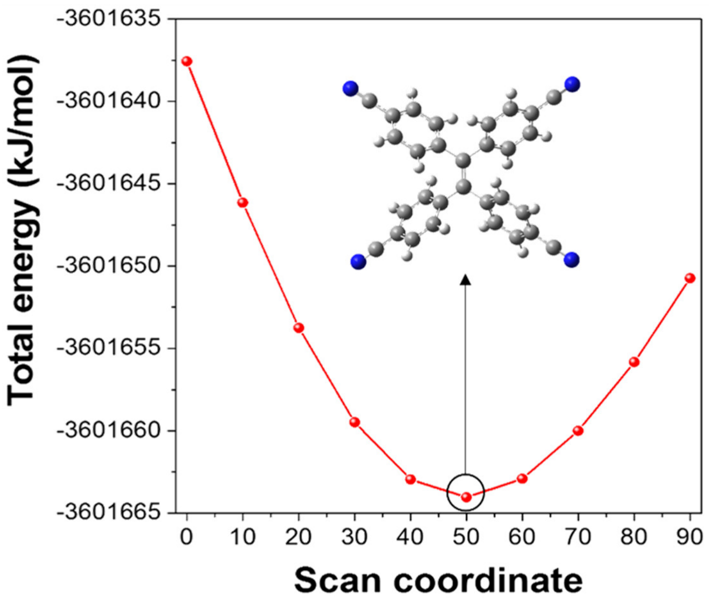

Supplementary Figure 6. Ground energy profile with different dihedral angles of 4CN in a gaseous environment by DFT calculation.

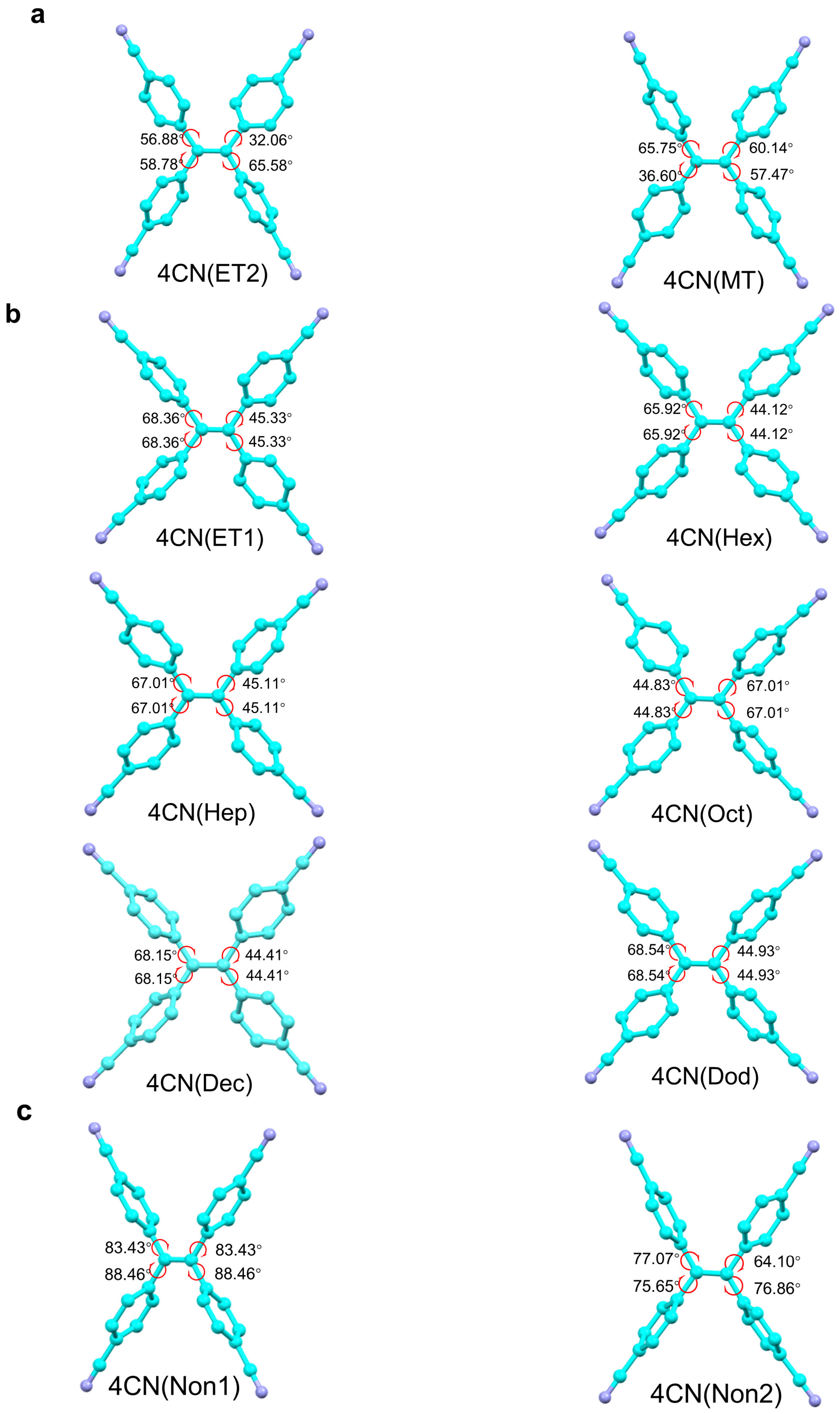
a
56.88°
32.06°
58.78°
65.58°
4CN(ET2)
65.75°
60.14°
36.60°
57.47°
4CN(MT)
b
68.36°
45.33°
68.36°
45.33°
4CN(ET1)
65.92°
44.12°
65.92°
44.12°
4CN(Hex)
67.01°
45.11°
67.01°
45.11°
4CN(Hep)
44.83°
67.01°
44.83°
67.01°
4CN(Oct)
68.15°
44.41°
68.15°
44.41°
4CN(Dec)
68.54°
44.93°
68.54°
44.93°
4CN(Dod)
c
83.43°
83.43°
88.46°
88.46°
4CN(Non1)
77.07°
64.10°
75.65°
76.86°
4CN(Non2)

d

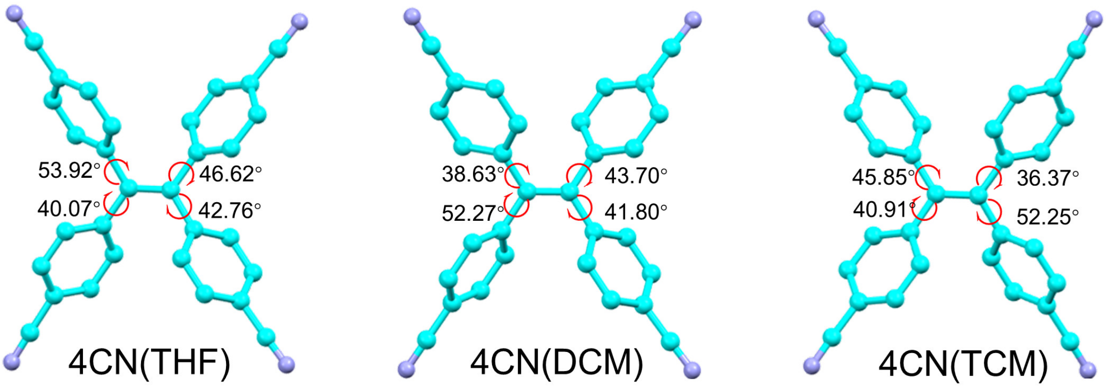

Supplementary Figure 7. a The dihedral angles between four phenyl groups and central ethenyl group of 4CN(ET2) and 4CN(MT) crystals. **b** The dihedral angles between four phenyl groups and central ethenyl group of 4CN(ET1)[1], 4CN(Hex), 4CN(Hep), 4CN(Oct), 4CN(Dec) and 4CN(Dod) crystals. **c** The dihedral angles between four phenyl groups and central ethenyl group of 4CN(Non1) and 4CN(Non2) crystals. **d** The dihedral angles between four phenyl groups and central ethenyl group of 4CN(THF), 4CN(DCM) and 4CN(TCM) crystals.

Supplementary Table 1. Summary of the dihedral angles (θ) between four phenyl groups and central ethenyl group of different crystals, average dihedral angles (θ_{aver}), and their corresponding maximum emission wavelength (λ_{em}), quantum yields (Φ) and lifetime (τ).

**Types**	θ_1	θ_2	θ_3	θ_4	θ_{aver}	λ_{em} **(nm)**	Φ	τ **(ns)**	**Group**
4CN(ET2)	65.58°	58.78°	56.88°	32.06°	53.32°	449	19.0%	2.1	**G1**
4CN(MT)	65.78°	60.11°	57.49°	36.56°	54.99°	449	12.5%	2.0	**G2**
4CN(ET1)	68.36°	68.36°	45.33°	45.33°	56.85°	470	37.5%	2.4	
4CN(Non2)	77.07°	76.86°	75.65°	64.10°	73.42°	471	16.6%	2.0	**G3**
4CN(Non1)	88.46°	88.46°	83.43°	83.43°	85.95°	482	44.8%	3.0	
4CN(DCM)	52.27°	43.70°	41.80°	38.63°	44.10°	481	21.6%	3.4	**G4**
4CN(TCM)	52.11°	45.74°	41.04°	36.43°	43.83°	497	65.4%	3.3	
4CN(THF)	53.57°	46.28°	42.91°	40.26°	45.76°	495	54.2%	3.3	
4CN(Hex)	65.92°	65.92°	44.12°	44.12°	55.02°	452,560	43.0%	1.2 (452 nm); 5.1 (560 nm)	**G2 white light**
4CN(Hep)	67.01°	67.01°	45.11°	45.11°	56.06°	441,569	31.6%	1.3 (441 nm); 5.1 (569 nm)	
4CN(Oct)	68.08°	68.08°	44.83°	44.83°	56.46°	441,571	28.6%	1.3 (441 nm); 4.5 (571 nm)	
4CN(Dec)	68.15°	68.15°	44.41°	44.41°	56.28°	460,569	27.6%	1.4 (460 nm); 4.0 (569 nm)	
4CN(Dod)	68.54°	68.54°	44.93°	44.93°	56.74°	469,549	27.2%	1.6 (469 nm); 4.6 (549 nm)	

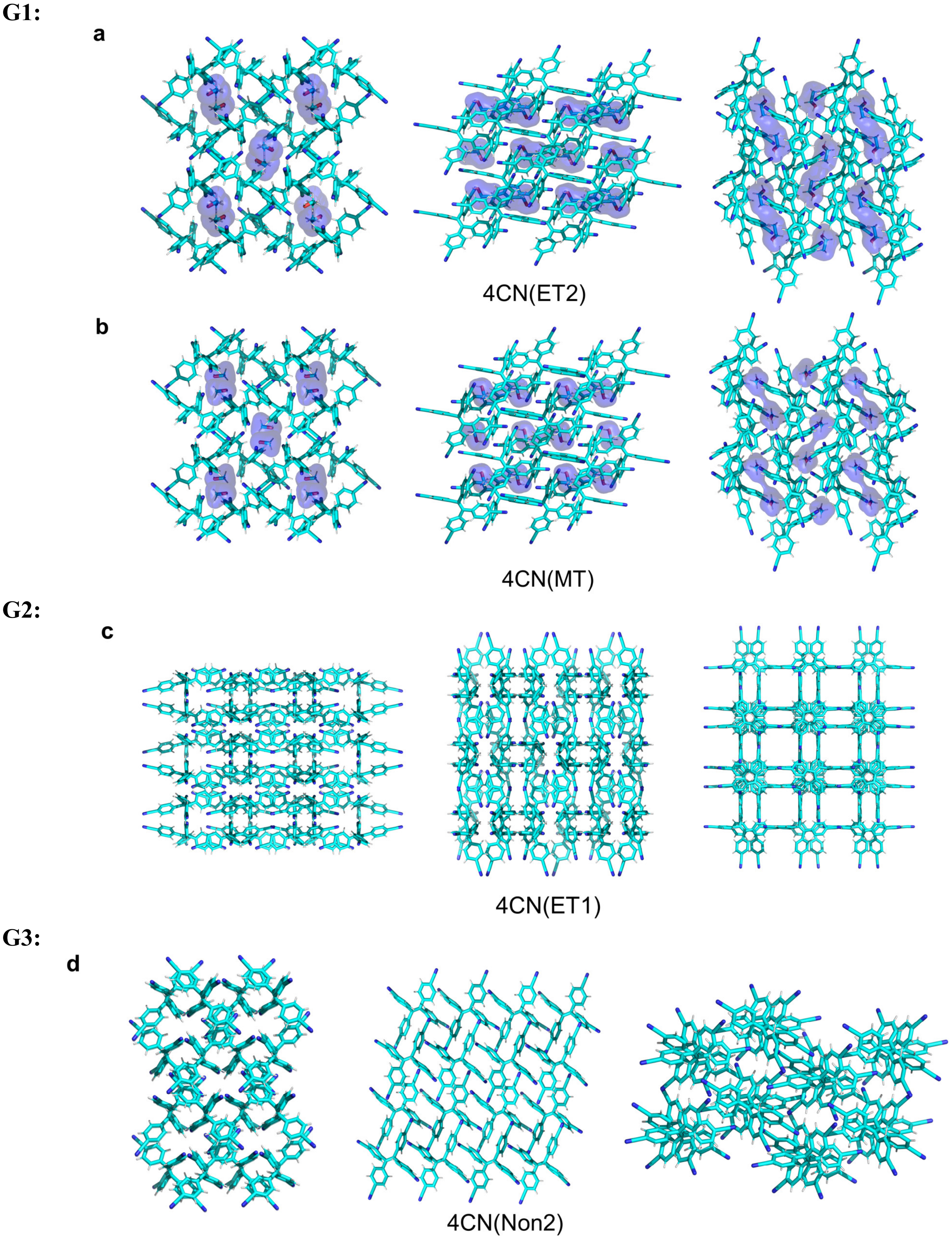
G1:
a
4CN(ET2)
b
4CN(MT)
G2:
c
4CN(ET1)
G3:
d
4CN(Non2)

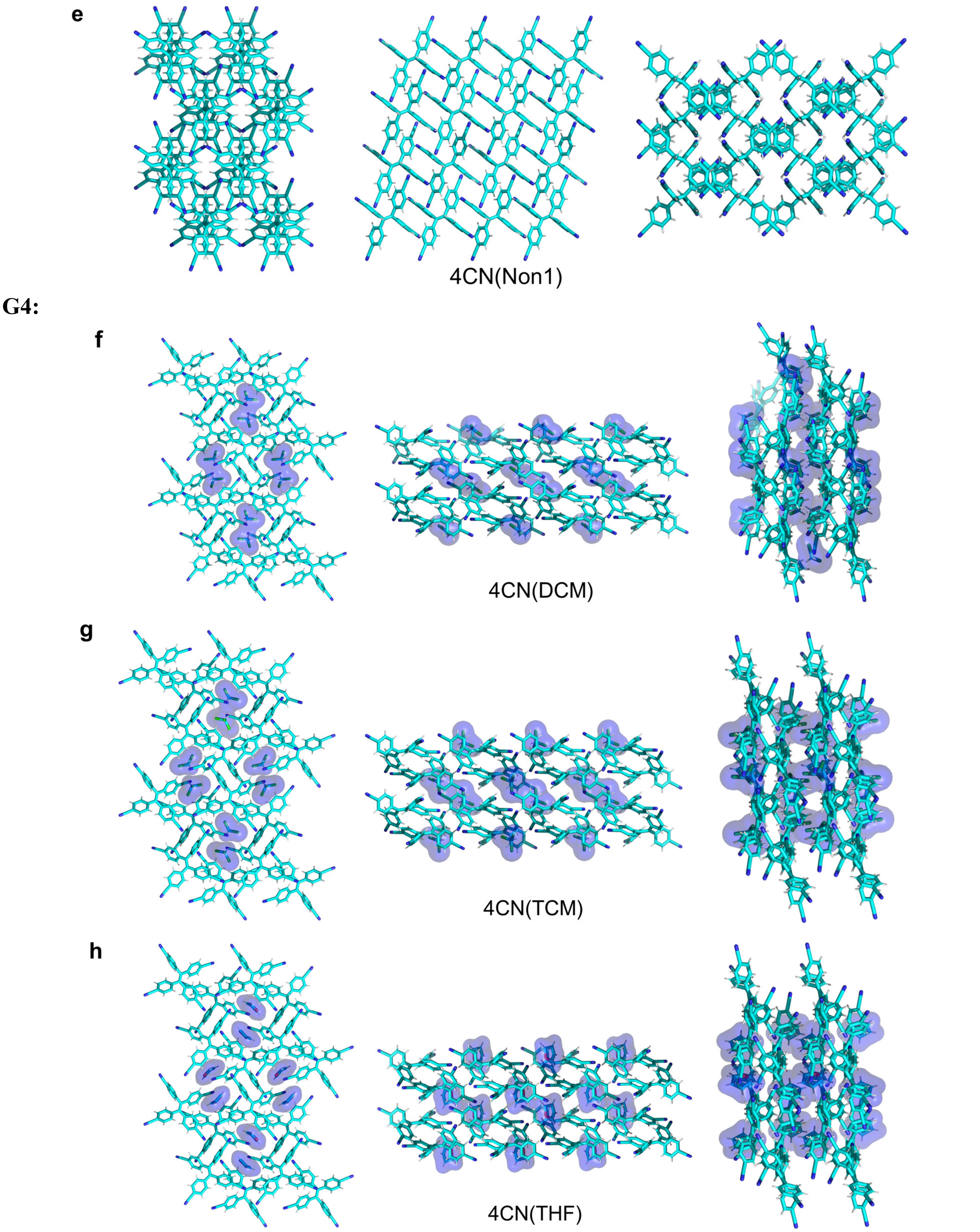

Supplementary Figure 8. Packing diagrams of **a** 4CN(ET2), **b** 4CN(MT), **c** 4CN(ET1)[1], **d** 4CN(Non2), **e** 4CN(Non1), **f** 4CN(DCM), **g** 4CN(TCM) and **h** 4CN(THF) along the a axis, b axis, and c axis (from left to right). The light blue region with space filling indicates the included solvents.

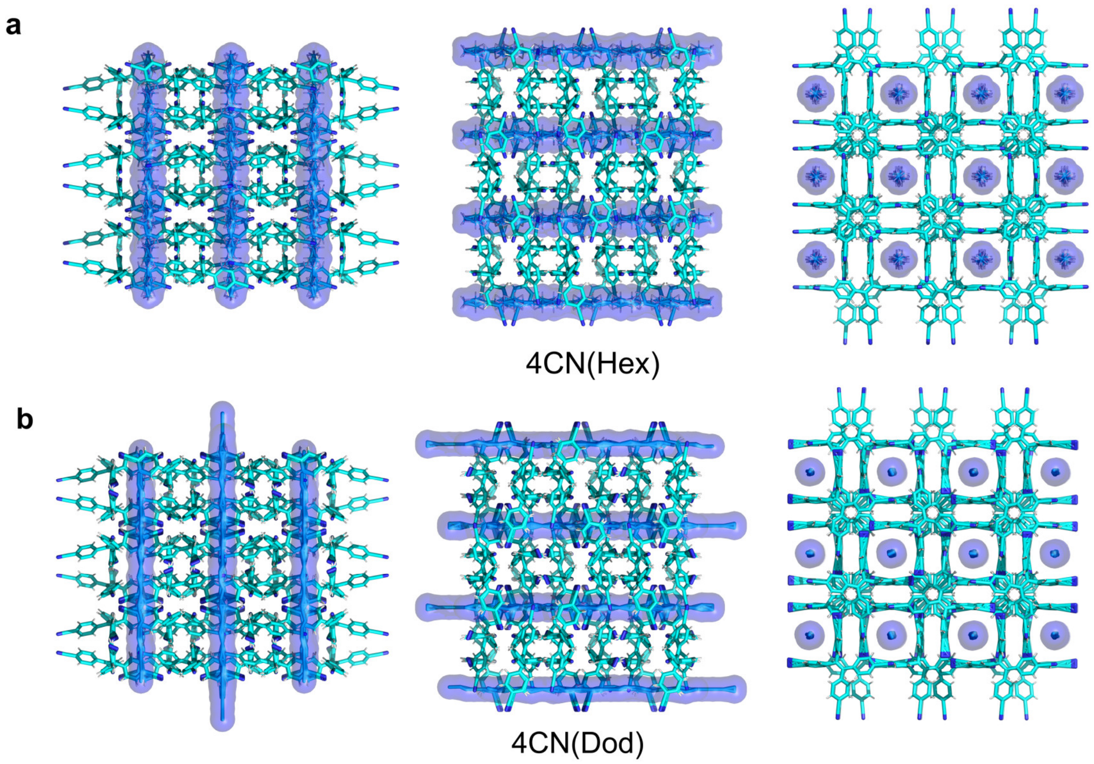

Supplementary Figure 9. Packing diagrams of **a** 4CN(Hex), **b** 4CN(Dod) (It should be noted that the packing diagrams of 4CN(Hep), 4CN(Oct) and 4CN(Dec) are similar to 4CN(Hex) and 4CN(Dod). Solvent molecules are disordered which cannot be displayed well in the stack). The light blue region with space filling indicates the included solvents.

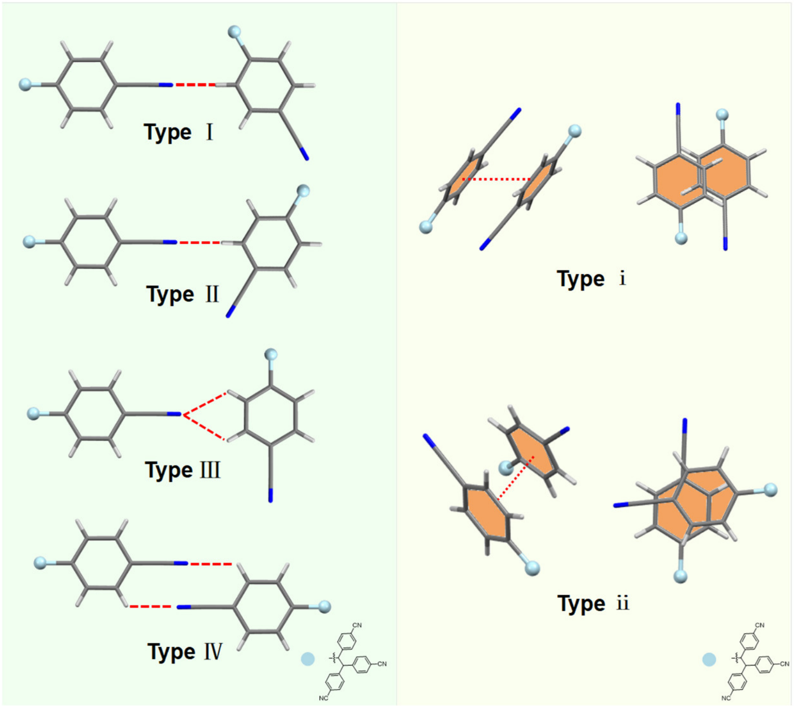

Supplementary Figure 10. Four typical C-H···N hydrogen bonds (left) and two typical π-π stacking interactions (right) in benzonitriles.

G1:

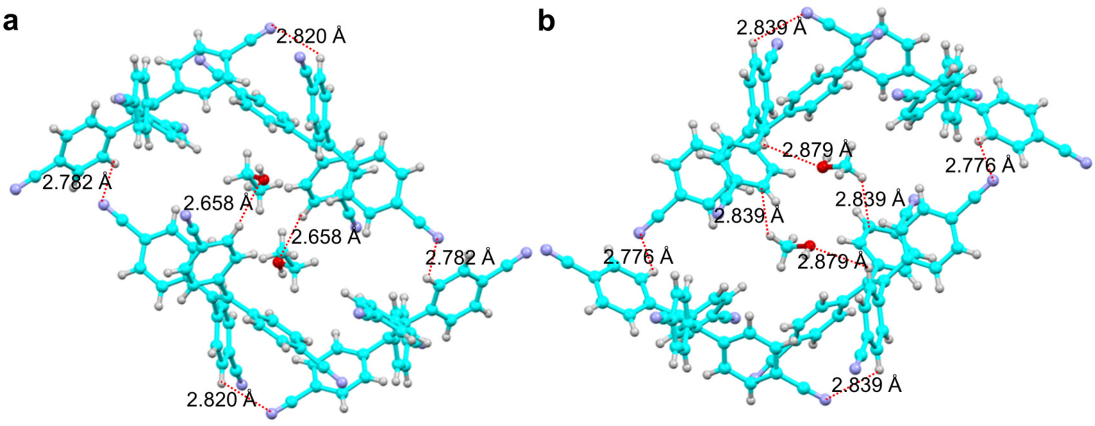

4CN(ET2) 4CN(MT)

G3:

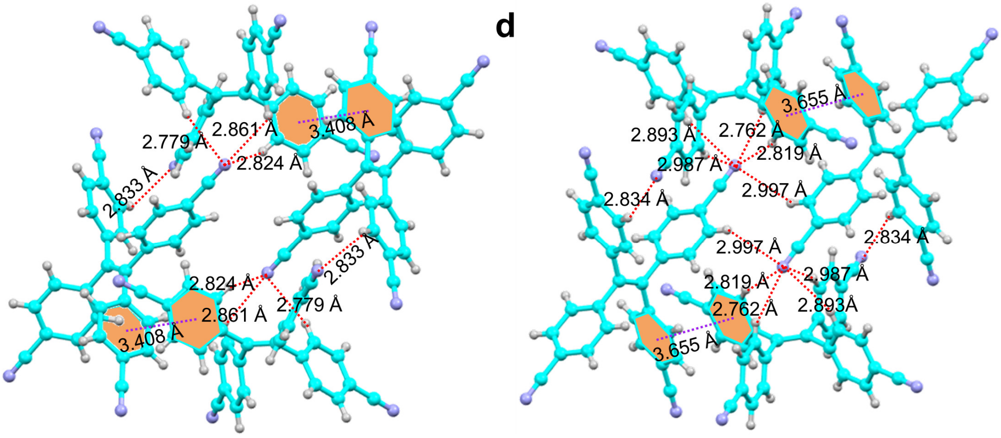

4CN(Non2) 4CN(Non1)

G2:

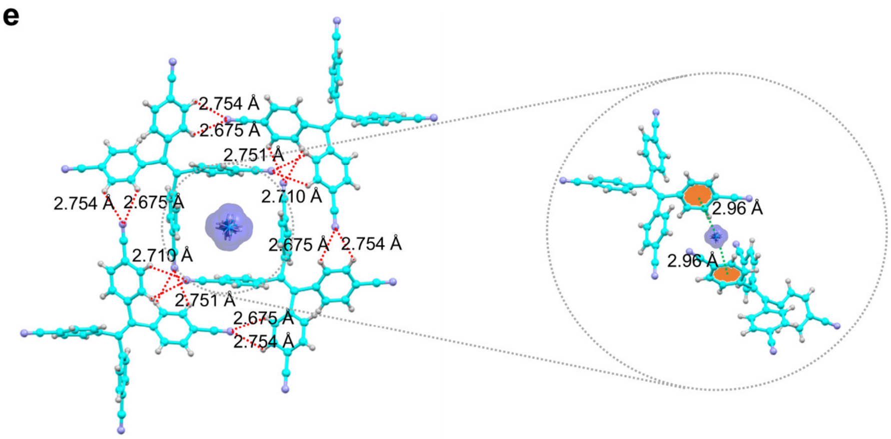

4CN(Hex)

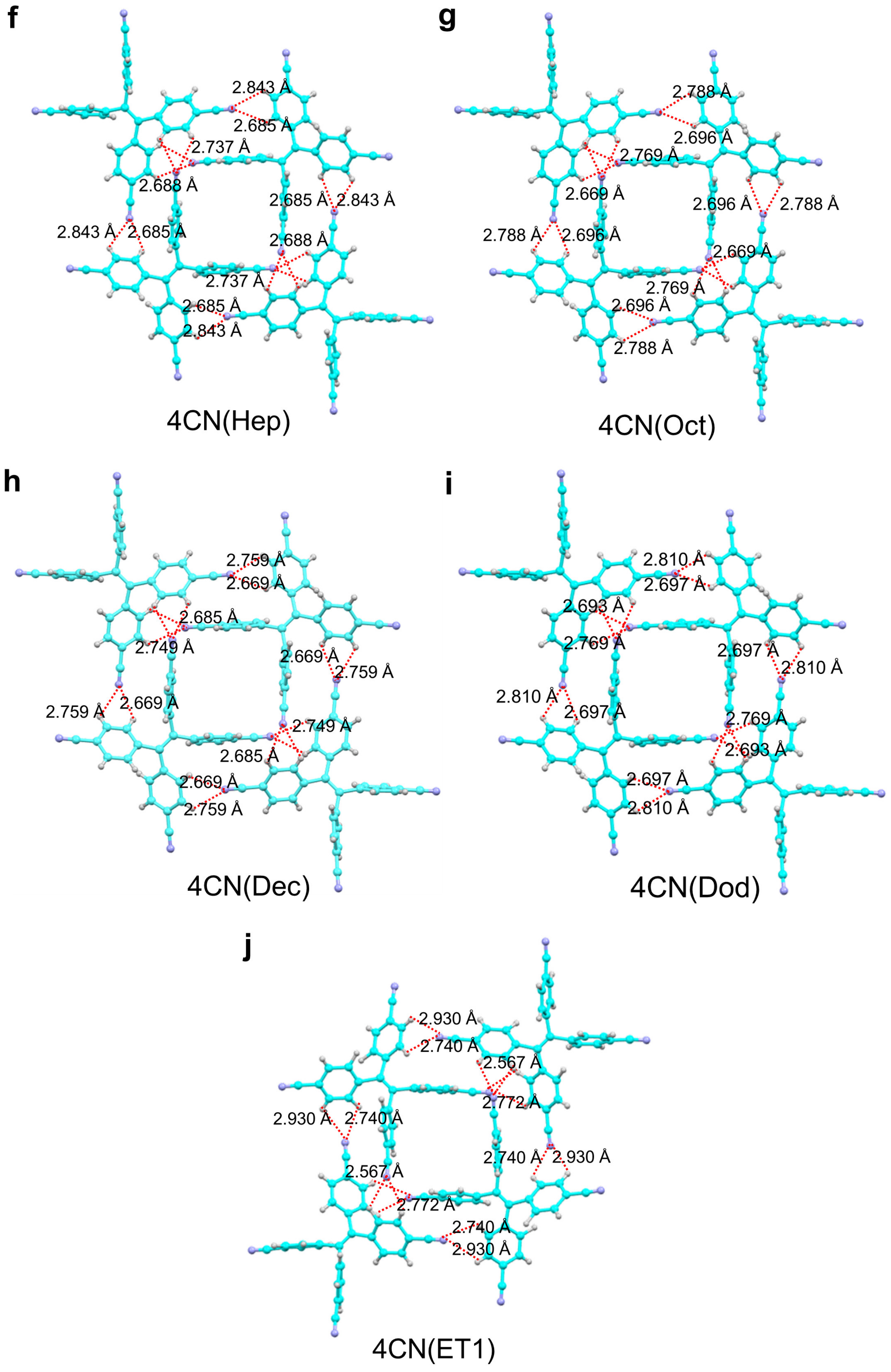
f
2.843 Å
2.685 Å
2.737 Å
2.688 Å
2.685 Å
2.843 Å
2.843 Å
2.685 Å
2.688 Å
2.737 Å
2.685 Å
2.843 Å
4CN(Hep)
g
2.788 Å
2.696 Å
2.769 Å
2.669 Å
2.696 Å
2.788 Å
2.788 Å
2.696 Å
2.669 Å
2.769 Å
2.696 Å
2.788 Å
4CN(Oct)
h
2.759 Å
2.669 Å
2.685 Å
2.749 Å
2.669 Å
2.759 Å
2.759 Å
2.669 Å
2.749 Å
2.685 Å
2.669 Å
2.759 Å
4CN(Dec)
i
2.810 Å
2.697 Å
2.693 Å
2.769 Å
2.697 Å
2.810 Å
2.810 Å
2.697 Å
2.769 Å
2.693 Å
2.697 Å
2.810 Å
4CN(Dod)
j
2.930 Å
2.740 Å
2.567 Å
2.772 Å
2.930 Å
2.740 Å
2.740 Å
2.930 Å
2.567 Å
2.772 Å
2.740 Å
2.930 Å
4CN(ET1)

G4:

k

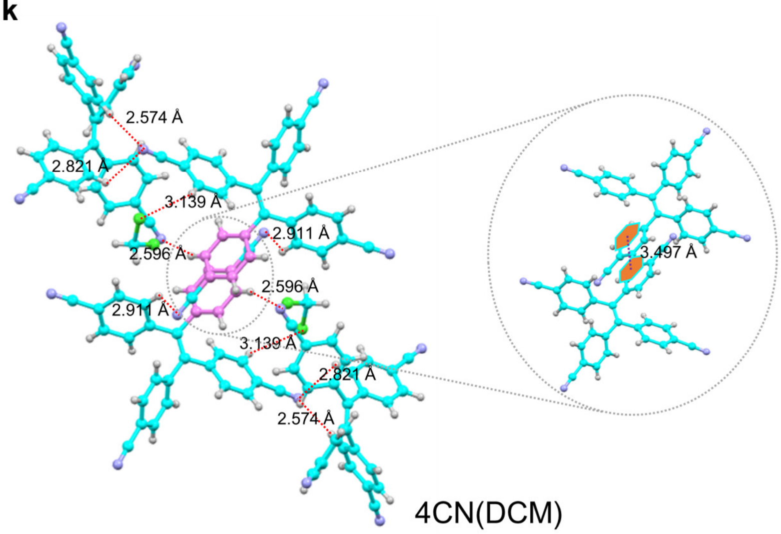

l

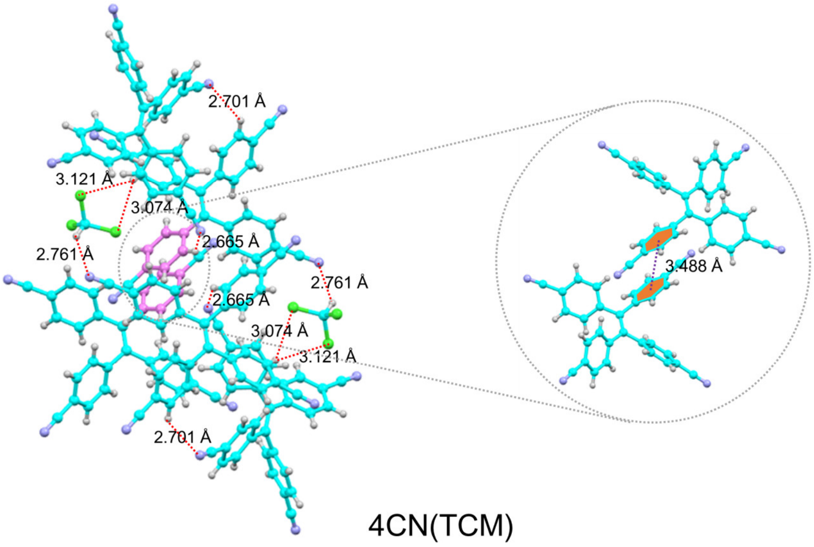

m

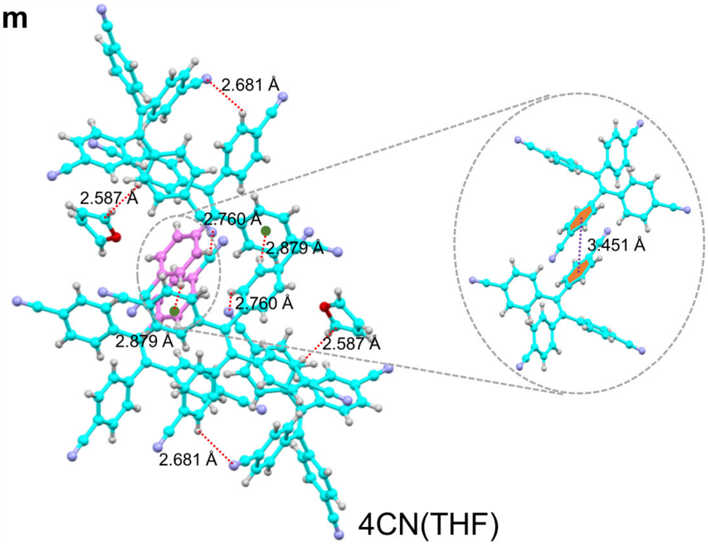

Supplementary Figure 11. Packing digrams of **a** 4CN(ET2) and **b** 4CN(MT), the red line indicates hydrogen bonding interactions (containing type I and type II C-H···N hydrogen bonds). **c** Packing digram of 4CN(Non2), the purple and red line indicates π-π (containing type ii π-π interactions) and hydrogen bonding interactions (containing type I and type II C-H···N hydrogen bonds), respectively. **d** Packing

digram of 4CN(Non1), the purple and red line indicates π-π (containing type ii π-π interactions) and hydrogen bonding interactions (containing type I, type II and type IV C-H···N hydrogen bonds), respectively. Packing digrams of **e** 4CN(Hex), **f** 4CN(Hep), **g** 4CN(Oct), **h** 4CN(Dec), **i** 4CN(Dod), and **j** 4CN(ET1), the red line indicates hydrogen bonding interactions (containing type III C-H···N hydrogen bonds). Inset: the green line indicates C-H···π interactions. Solvent molecules are so disordered that they do not appear in the crystal structures of 4CN(Hep), 4CN(Oct), 4CN(Dod) and 4CN(ET1). **k** Packing digram of 4CN(DCM), the red line indicates hydrogen bonding interactions (containing type II C-H···N hydrogen bonds). Inset: the purple line indicates π-π interactions (containing type I π-π interactions). **l** Packing digram of 4CN(TCM), the red line indicates hydrogen bonding interactions (containing type I and type II C-H···N hydrogen bonds). Inset: the purple line indicates π-π interactions (containing type i π-π interactions). **m** Packing digram of 4CN(THF), the green and red line indicates C-H···π and hydrogen bonding interactions (containing type I and type II C-H···N hydrogen bonds), respectively. Inset: the purple line indicates π-π interactions (containing type i π-π interactions).

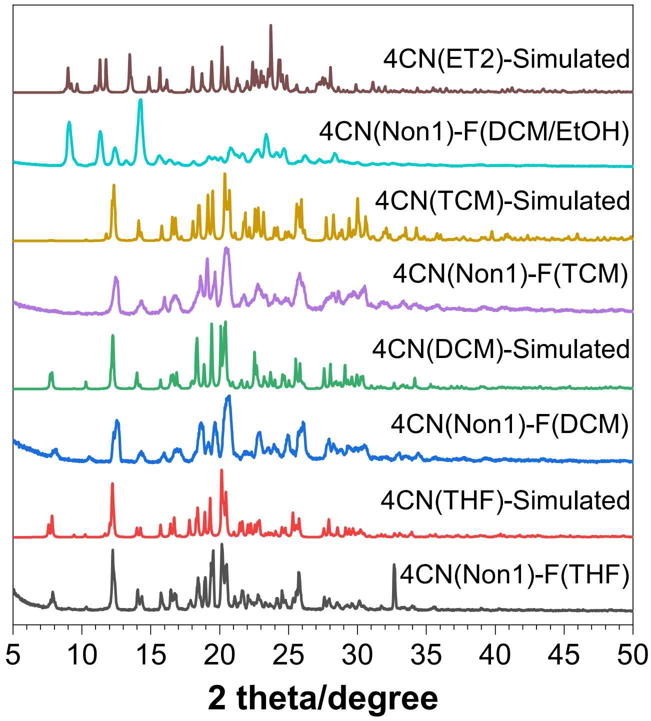

Supplementary Figure 12. The PXRD spectra of 4CN(Non1) crystals with different solvents fuming and simulated PXRD of 4CN(THF), 4CN(DCM), 4CN(TCM) and 4CN(ET2).

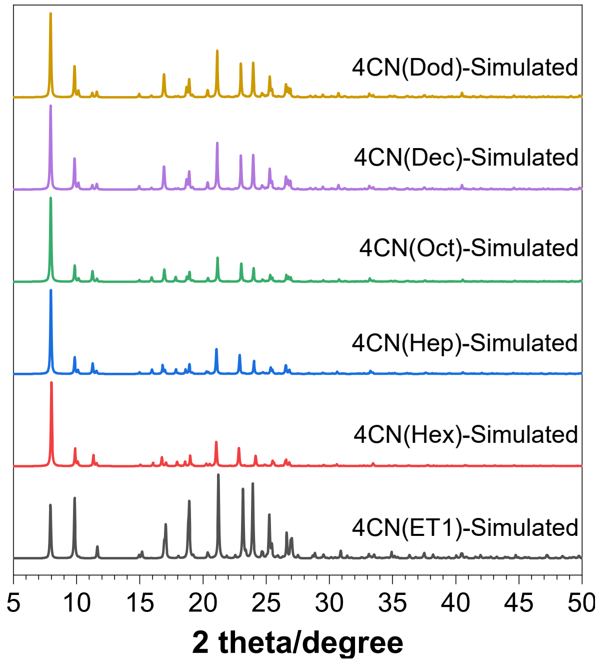

Supplementary Figure 13. The PXRD spectra of simulated 4CN(ET1), 4CN(Hex), 4CN(Hep), 4CN(Oct), 4CN(Dec) and 4CN(Dod).

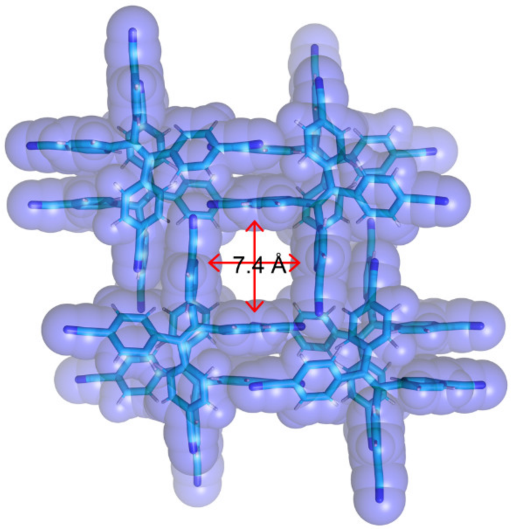

Supplementary Figure 14. Packing diagram of 4CN(ET1) along the [001] direction. The dimensions of the cavity are highlighted by red arrows.

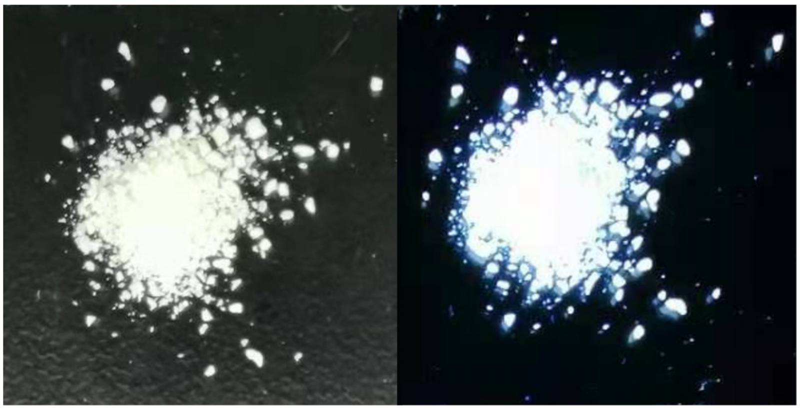

Supplementary Figure 15. The photographs of 4CN(Hex) crystals under daylight (left) and UV light (right).

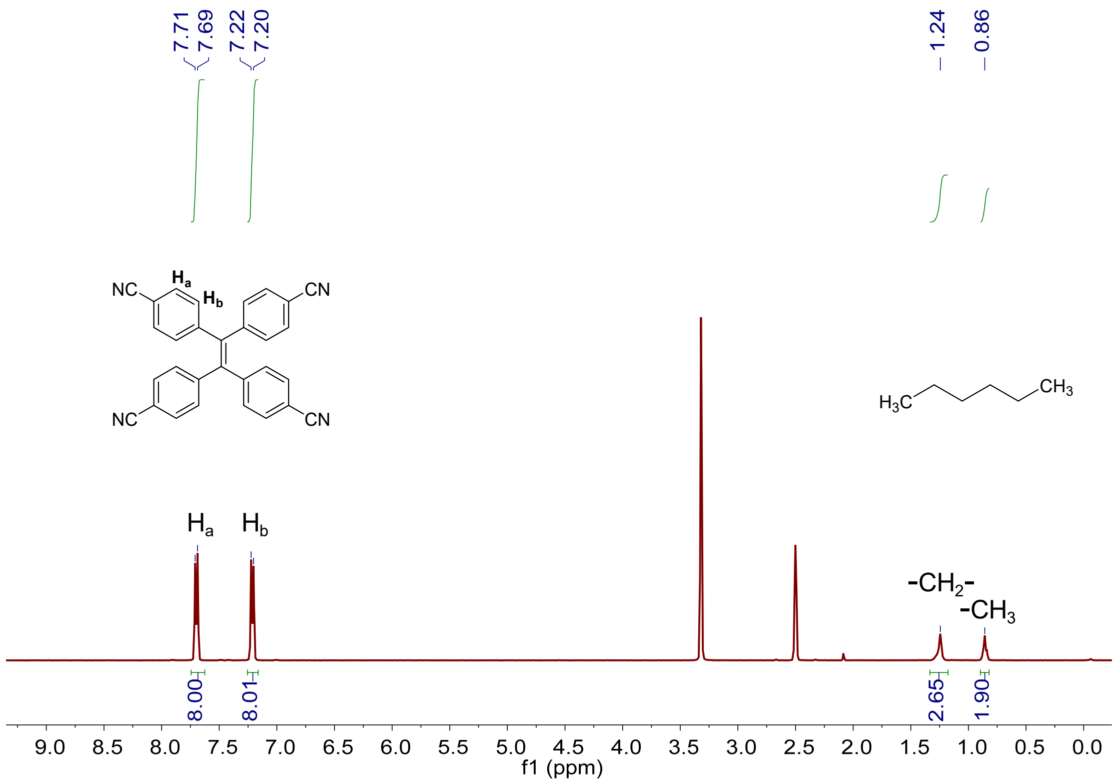

Supplementary Figure 16. ^{1}H NMR (400 MHz, 298 K) spectra of 4CN(Hex) in DMSO-d_6.

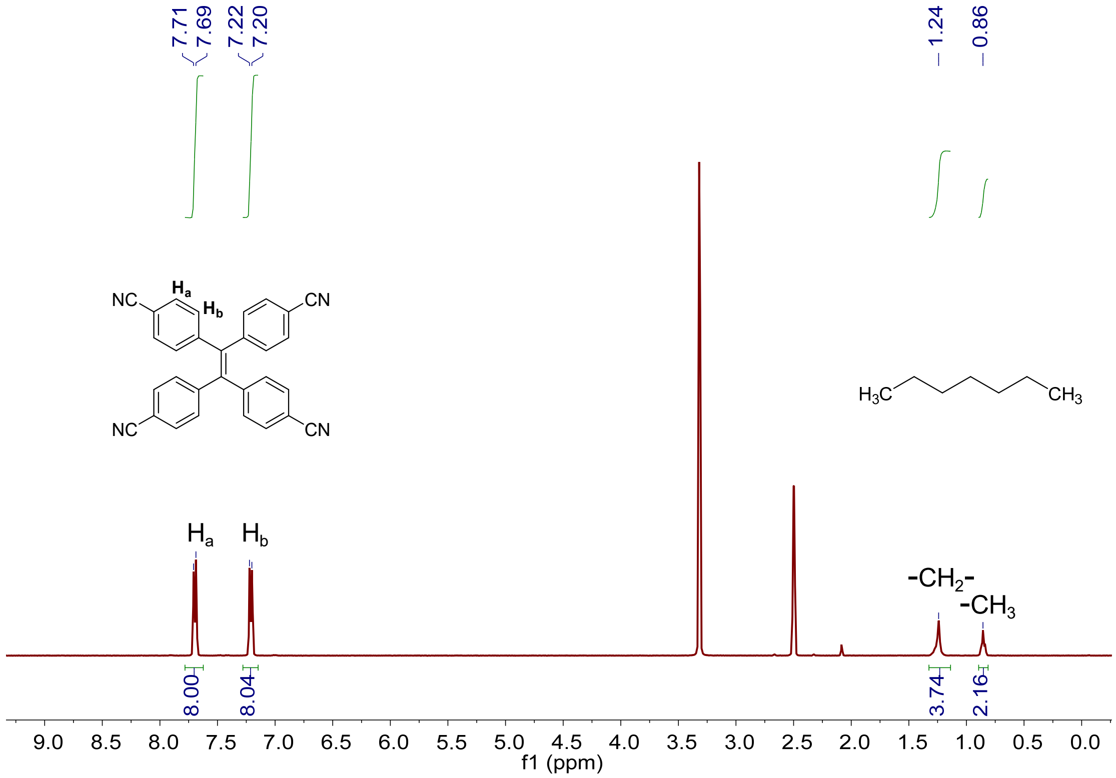

Supplementary Figure 17. ^{1}H NMR (400 MHz, 298 K) spectra of 4CN(Hep) in DMSO-d_6.

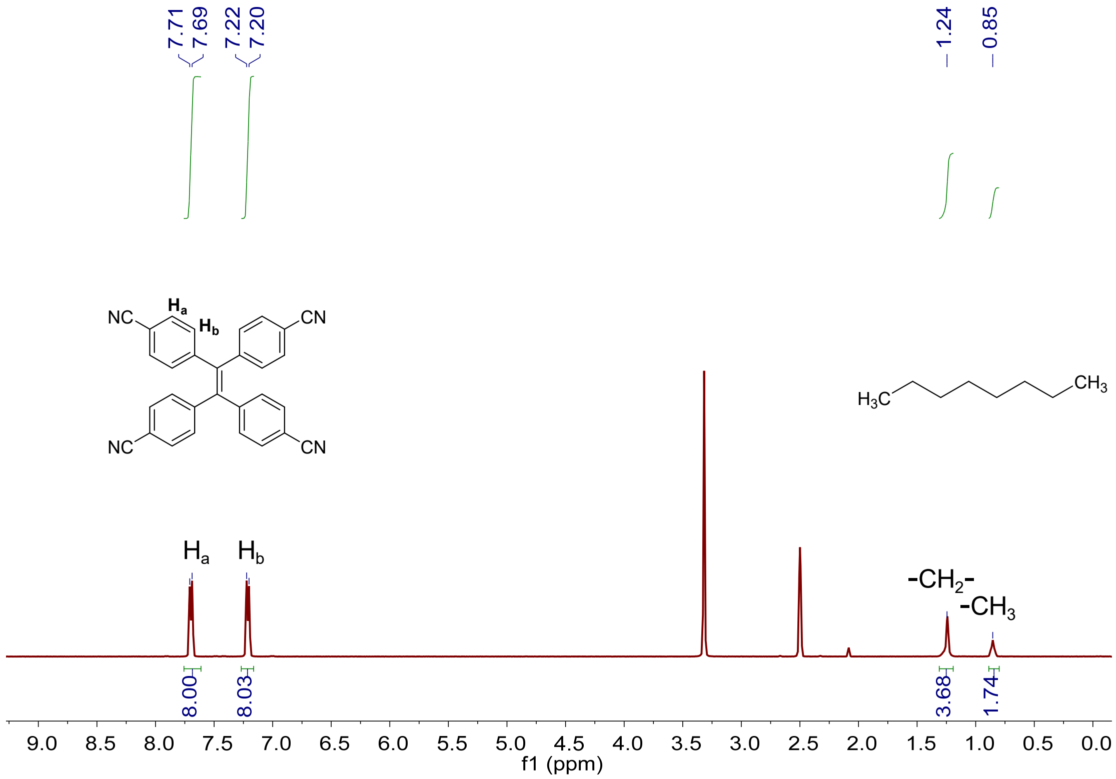

Supplementary Figure 18. ^{1}H NMR (400 MHz, 298 K) spectra of 4CN(Oct) in DMSO-d_6.

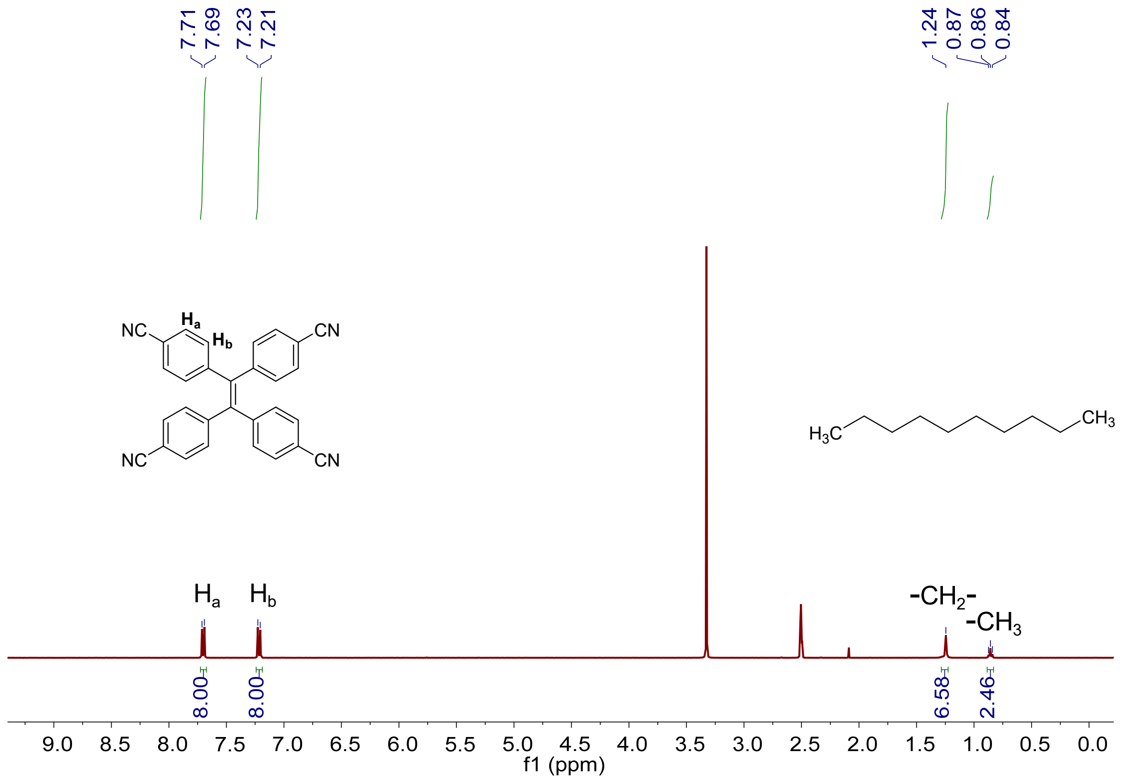

Supplementary Figure 19. ^{1}H NMR (400 MHz, 298 K) spectra of 4CN(Dec) in DMSO-d_6.

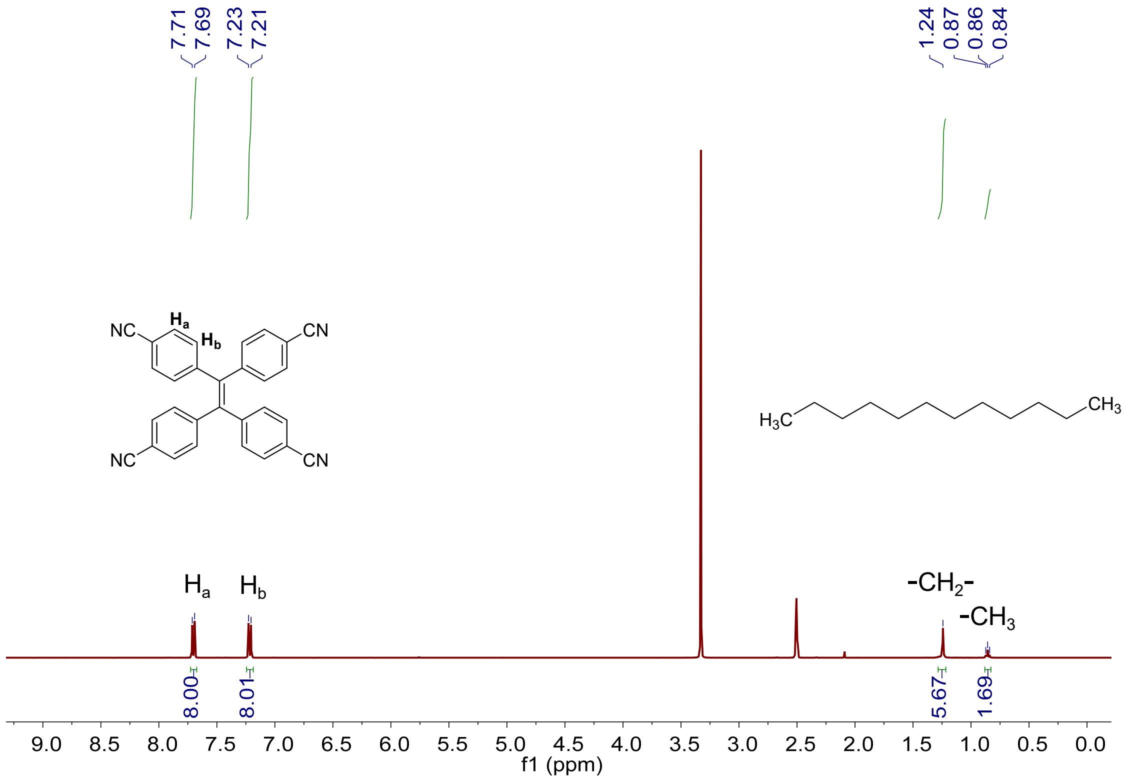

Supplementary Figure 20. ^{1}H NMR (400 MHz, 298 K) spectra of 4CN(Dod) in DMSO-d_6.

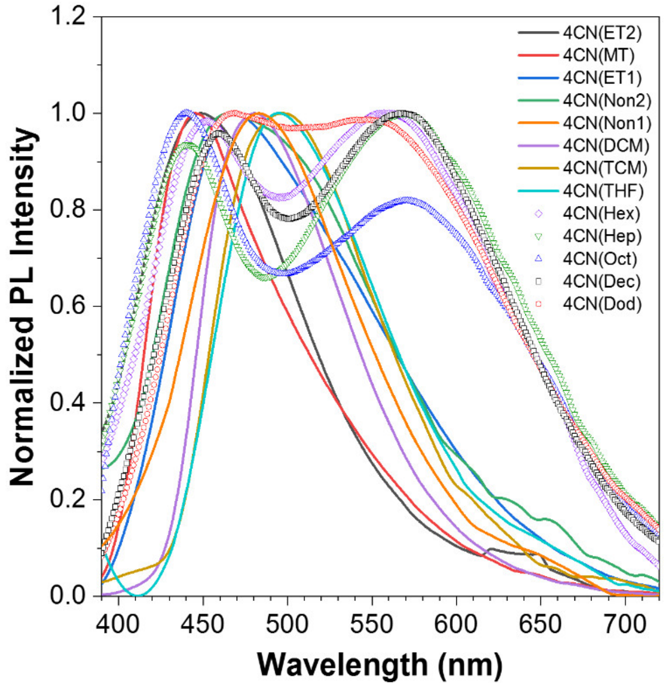

Supplementary Figure 21. Normalized fluorescence spectra of thirteen 4CN crystals.

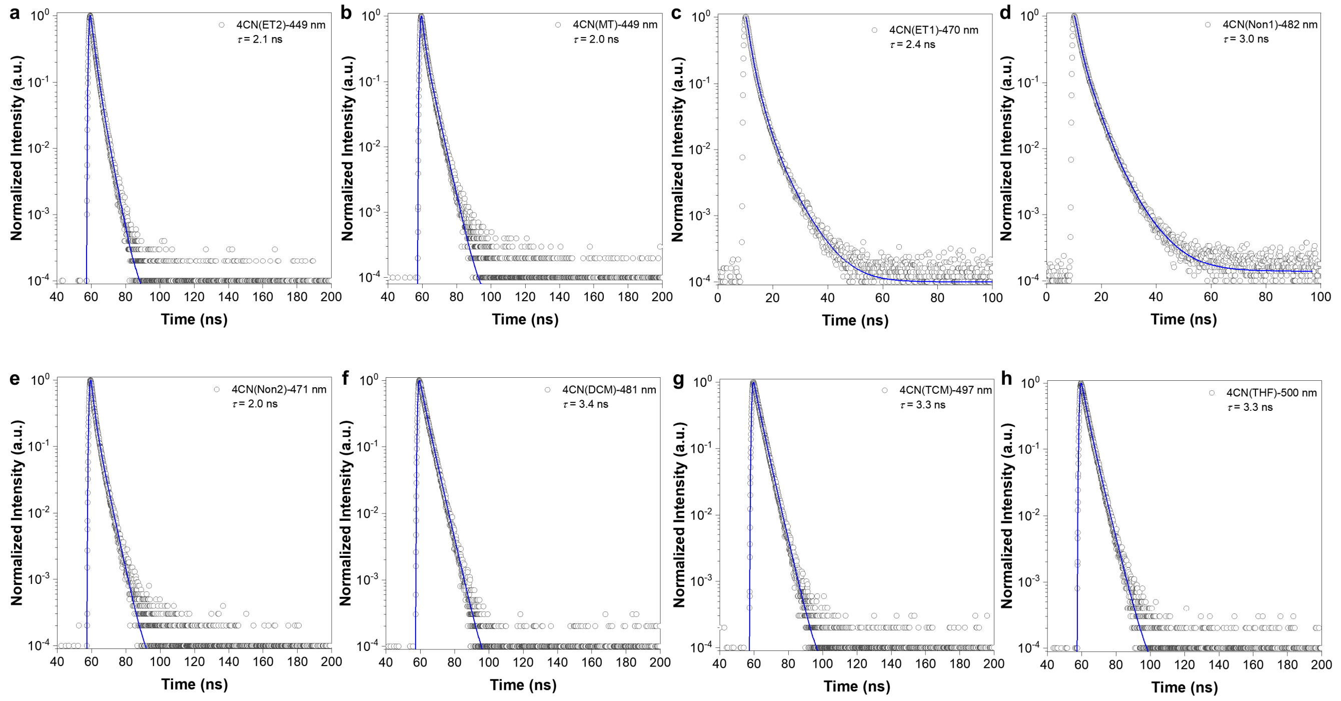

Supplementary Figure 22. Fluorescence decay curves of non-white-emitting **a** 4CN(ET2), **b** 4CN(MT), **c** 4CN(ET1), **d** 4CN(Non1), **e** 4CN(Non2), **f** 4CN(DCM), **g** 4CN(TCM) and **h** 4CN(THF) crystals.

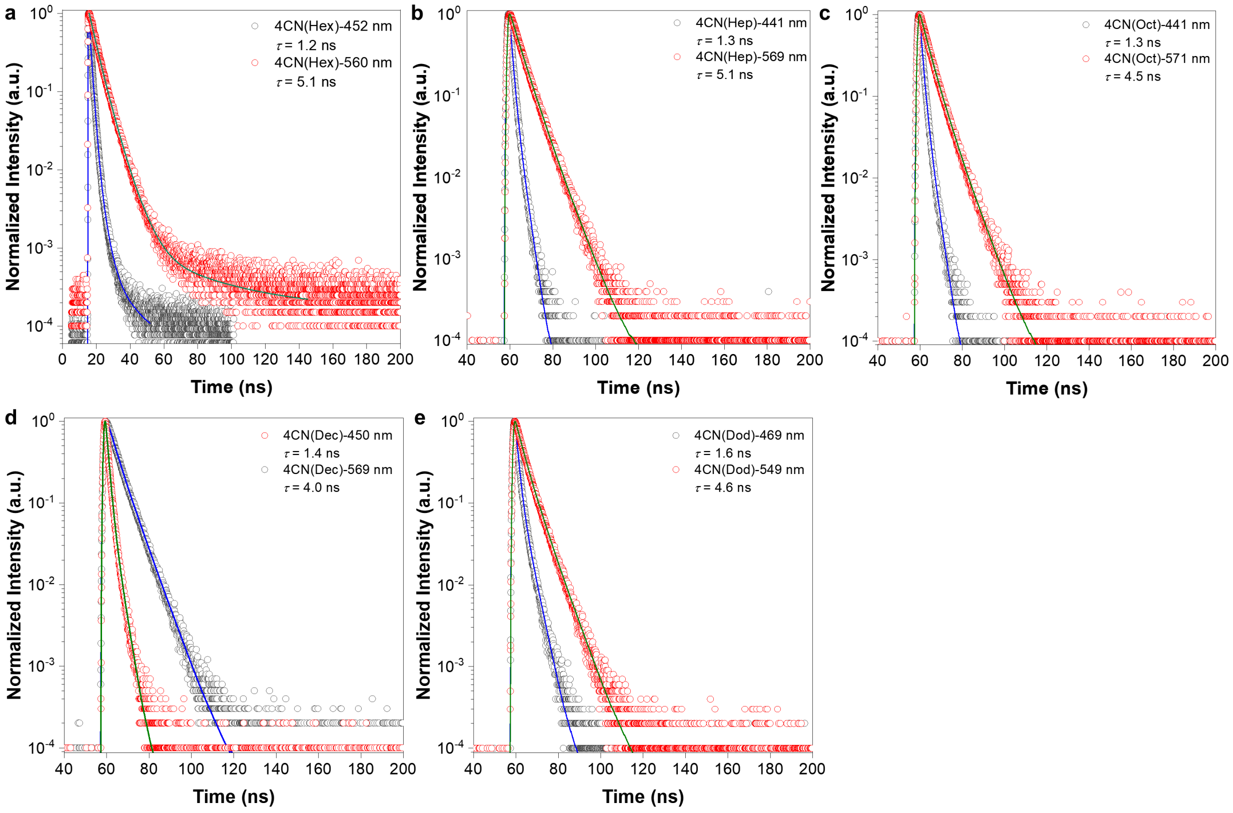

Supplementary Figure 23. Fluorescence decay curves of white-emitting **a** 4CN(Hex), **b** 4CN(Hep), **c** 4CN(Oct), **d** 4CN(Dec) and **e** 4CN(Dod) crystals.

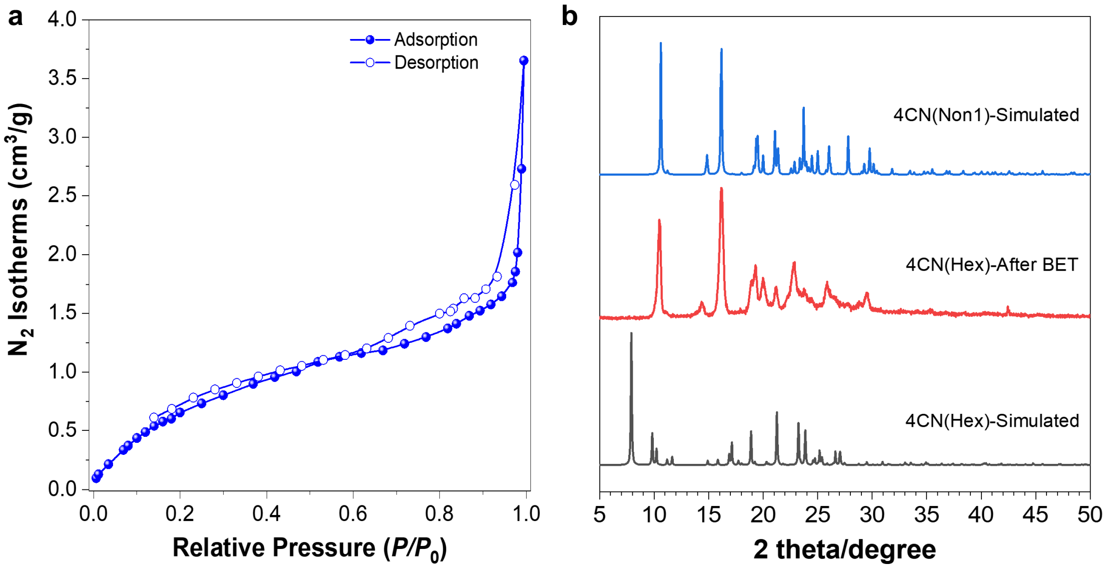

Supplementary Figure 24. **a** Sorption isotherms measured on 4CN(Hex) for N_2 at 77 K. **b** The PXRD spectra of simulated 4CN(Hex), 4CN(Non1) and 4CN(Hex) after gas adsorption-desorption.

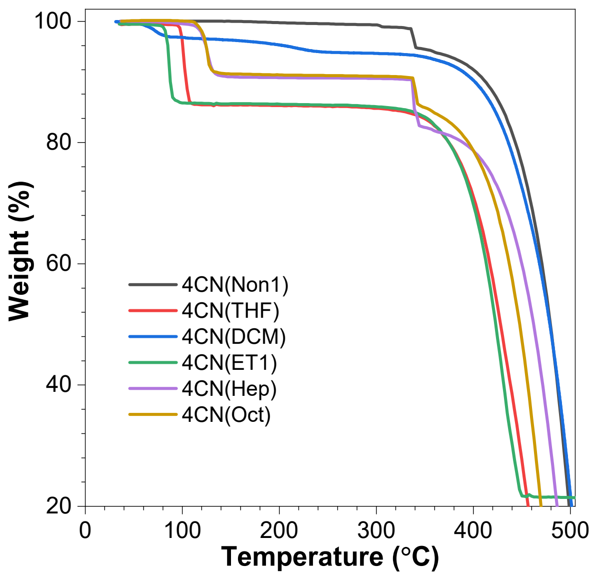

Supplementary Figure 25. TGA scan of 4CN(Non1), 4CN(THF), 4CN(DCM), 4CN(ET1), 4CN(Hep) and 4CN(Oct) under nitrogen atmosphere at a heating rate of 10 °C/min.

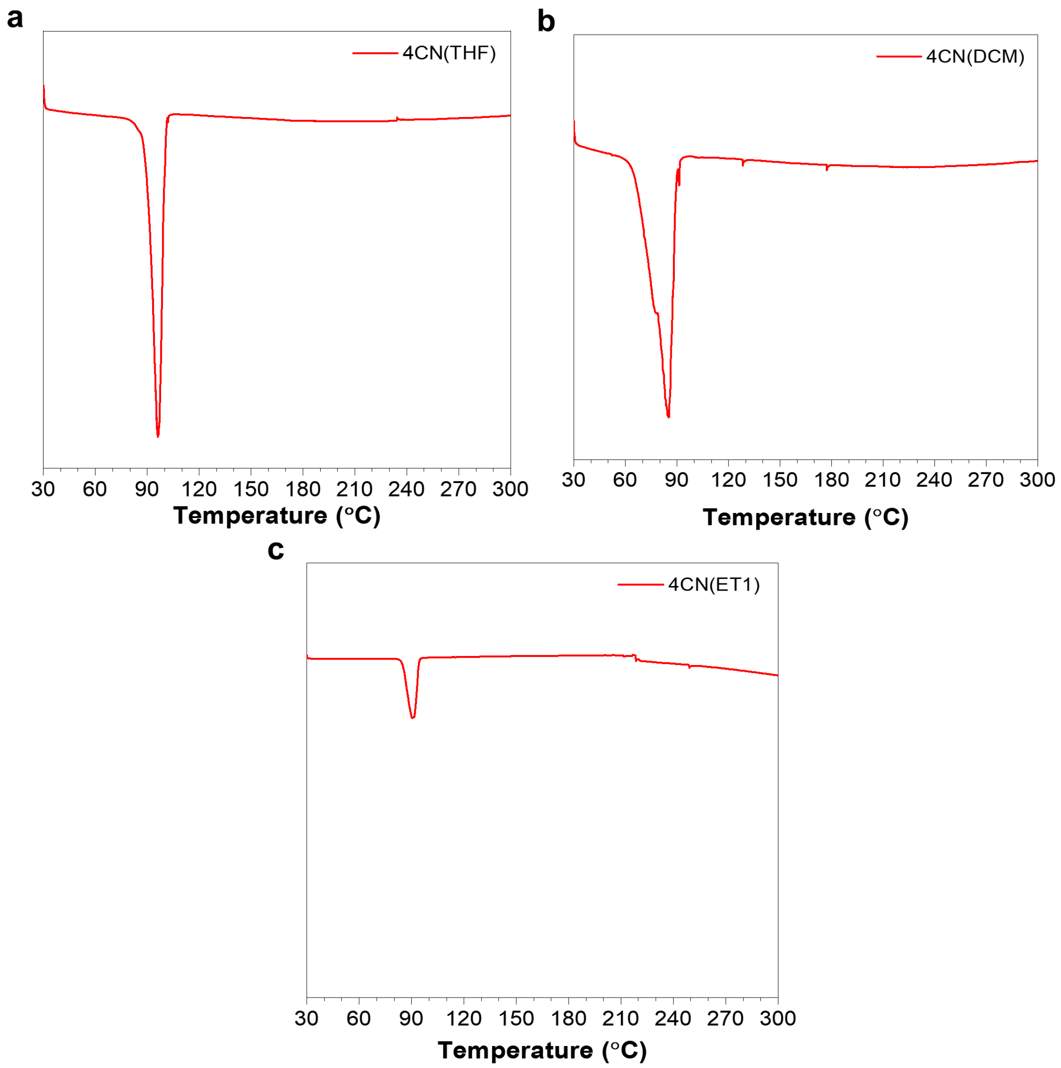

Supplementary Figure 26. Differential scanning calorimetry (DSC) of **a** 4CN(THF), **b** 4CN(DCM) and **c** 4CN(ET1) crystals at a heating rate of 5 °C/min.

Conversion from 4CN(MT), 4CN(ET2), 4CN(DCM), 4CN(TCM) and 4CN(THF) to 4CN(Non1)

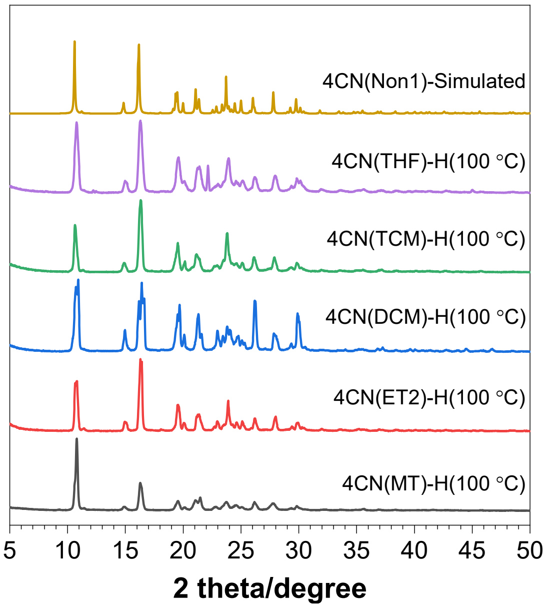

Supplementary Figure 27. The PXRD spectra of simulated 4CN(Non1), heated 4CN(MT), heated 4CN(ET2), heated 4CN(DCM), heated 4CN(TCM) and 4CN(THF) crystals.

Conversion from 4CN(ET1) (G2), 4CN(ET2) (G1) to 4CN(Non1) (G3)

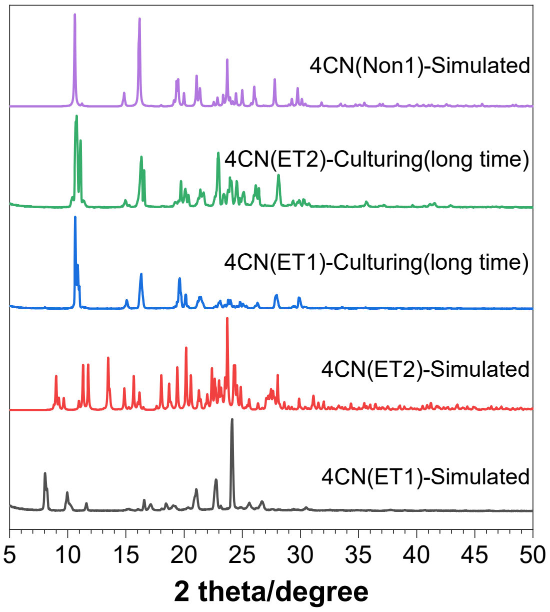

Supplementary Figure 28. The PXRD spectra of simulated 4CN(ET1), 4CN(ET2), 4CN(Non1), and 4CN(ET1), 4CN(ET2) crystals after culturing in solvents for long time.

Reversible conversion between 4CN(ET2) (G1) and 4CN(Non1) (G3)

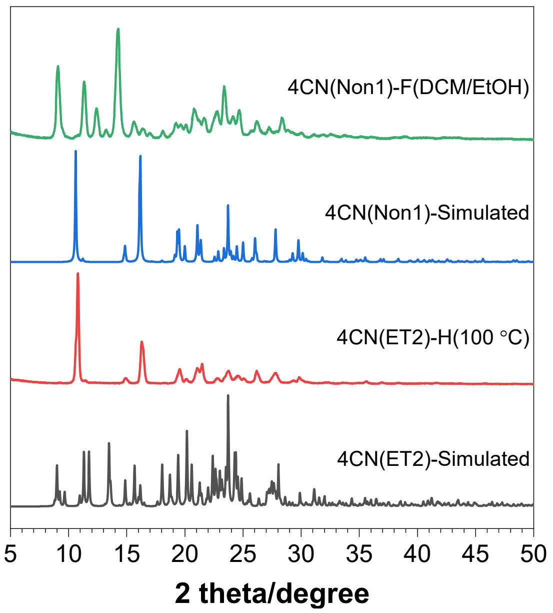

Supplementary Figure 29. The PXRD spectra of simulated 4CN(ET2) and 4CN(Non1), heated 4CN(ET2), fumed (dichloromethane/ethanol vapor) 4CN(Non1) crystals.

Reversible conversion between 4CN(THF) (G4) and 4CN(Non1) (G3)

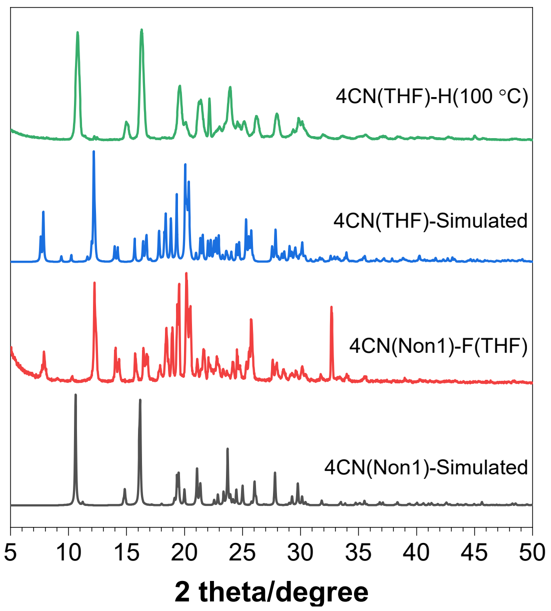

Supplementary Figure 30. The PXRD spectra of simulated 4CN(Non1) and 4CN(THF), fumed (tetrahydrofuran vapor) 4CN(Non1), heated 4CN(THF) crystals.

Reversible conversion between 4CN(DCM) (G4) and 4CN(Non1) (G3)

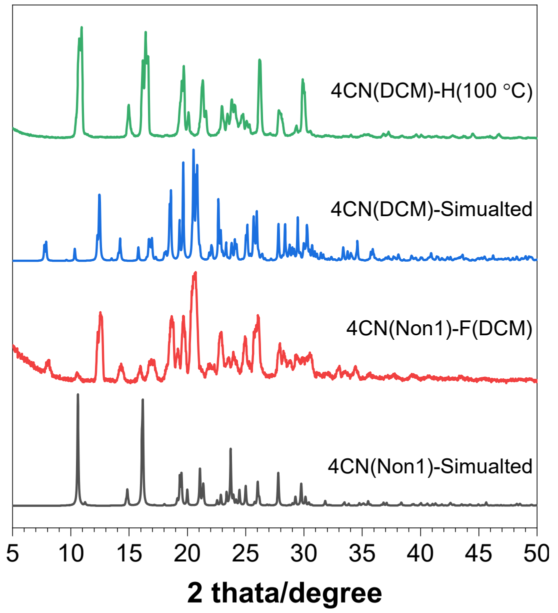

Supplementary Figure 31. The PXRD spectra of simulated 4CN(Non1) and 4CN(DCM), fumed (dichloromethane vapor) 4CN(Non1), heated 4CN(DCM) crystals.

Reversible conversion between 4CN(TCM) (G4) and 4CN(Non1) (G3)

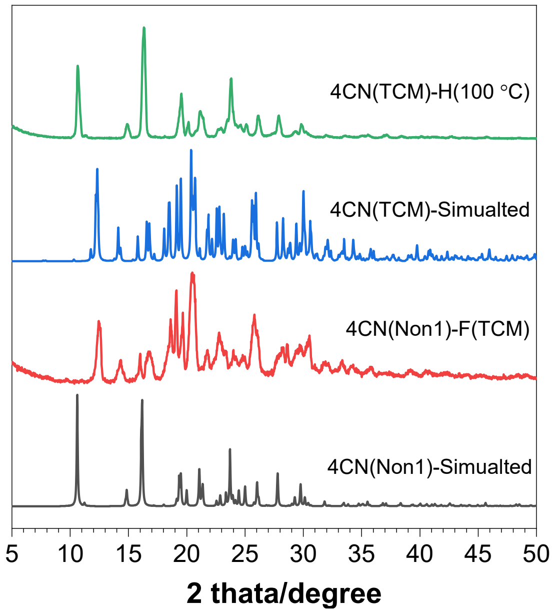

Supplementary Figure 32. The PXRD spectra of simulated 4CN(Non1) and 4CN(TCM), fumed (chloroform vapor) 4CN(Non1), heated 4CN(TCM) crystals.

Reversible conversion between 4CN(THF) (G4) and 4CN(ET2) (G1)

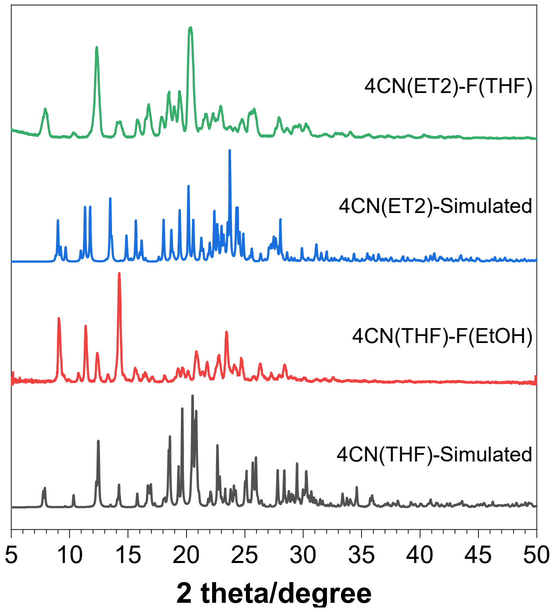

Supplementary Figure 33. The PXRD spectra of simulated 4CN(THF) and 4CN(ET2), fumed (ethanol vapor) 4CN(THF), fumed (tetrahydrofuran vapor) 4CN(ET2) crystals.

Reversible conversion between 4CN(THF) (G4) and 4CN(Hex) (G2)

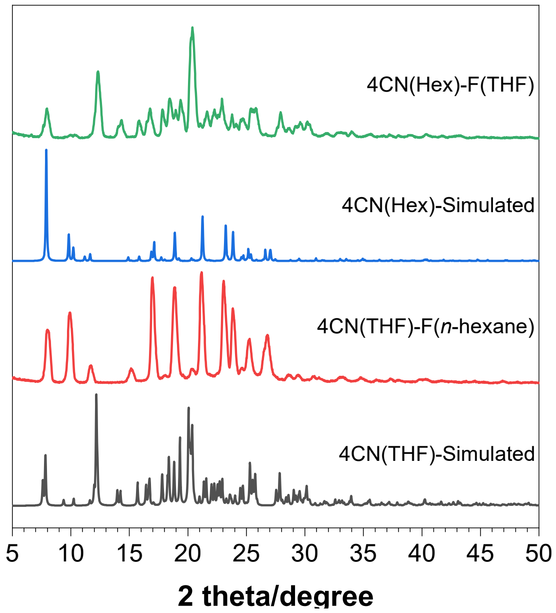

Supplementary Figure 34. The PXRD spectra of simulated 4CN(THF) and 4CN(Hex), fumed (*n*-hexane vapor) 4CN(THF), fumed (tetrahydrofuran vapor) 4CN(Hex) crystals.

Reversible conversion between 4CN(DCM) (G4) and 4CN(Hex) (G2)

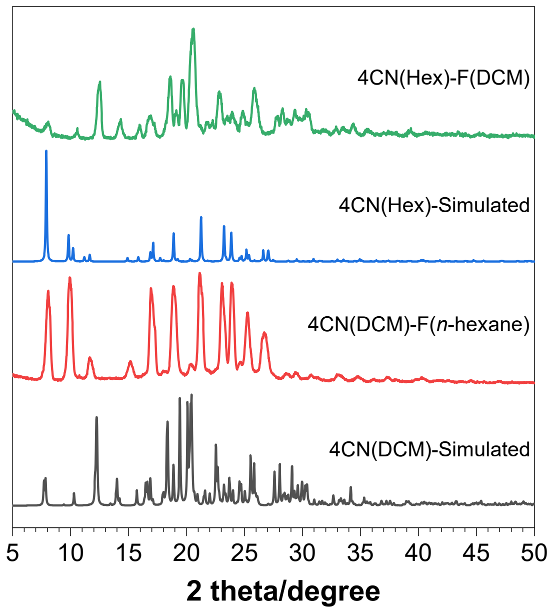

Supplementary Figure 35. The PXRD spectra of simulated 4CN(DCM) and 4CN(Hex), fumed (*n*-hexane vapor) 4CN(DCM), fumed (dichloromethane vapor) 4CN(Hex) crystals.

Reversible conversion between 4CN(DCM) (G4) and 4CN(ET2) (G1)

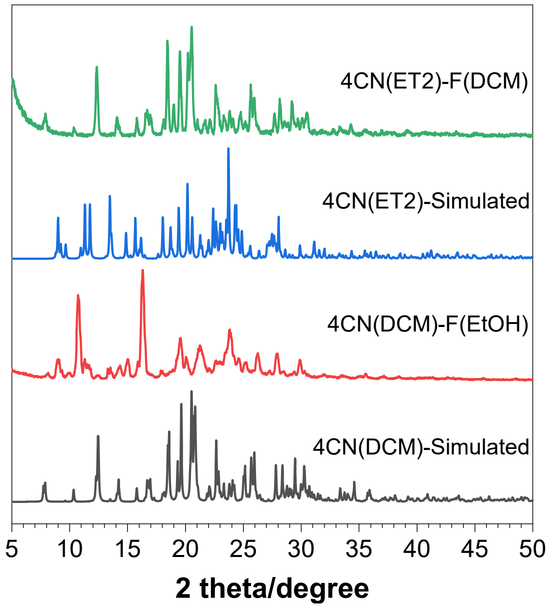

Supplementary Figure 36. The PXRD spectra of simulated 4CN(DCM) and 4CN(ET2), fumed (ethanol vapor) 4CN(DCM), fumed (dichloromethane vapor) 4CN(ET2) crystals.

Conversion from 4CN(ET1) (G2) to 4CN(ET2) (G1)

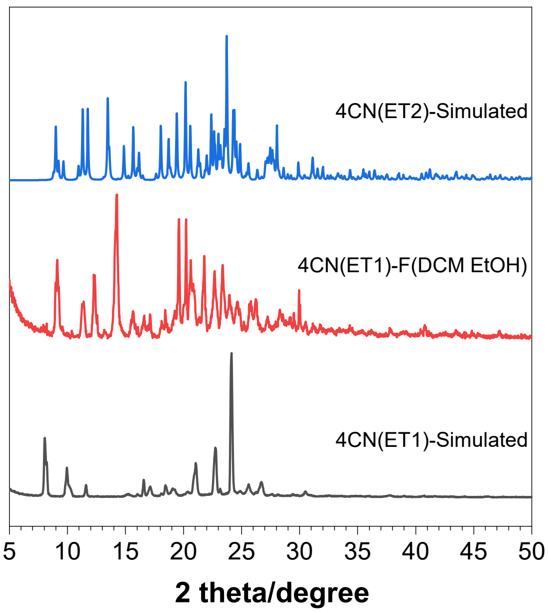

Supplementary Figure 37. The PXRD spectra of simulated 4CN(ET1) and 4CN(ET2), fumed (dichloromethane/ethanol mixed vapor) 4CN(ET1) crystals.

Conversion from 4CN(ET2) (G1) to 4CN(Hex) (G2)

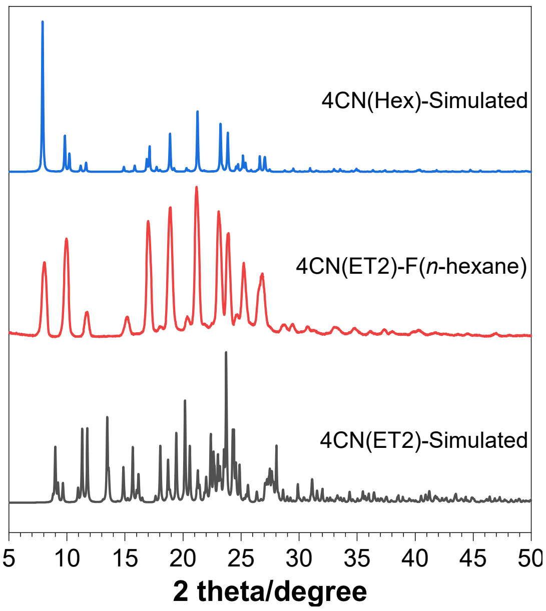

Supplementary Figure 38. The PXRD spectra of simulated 4CN(ET2) and 4CN(Hex), fumed (*n*-hexane vapor) 4CN(ET2) crystals.

Reversible conversion between 4CN(ET2) (G1) and 4CN(Non1) (G3)

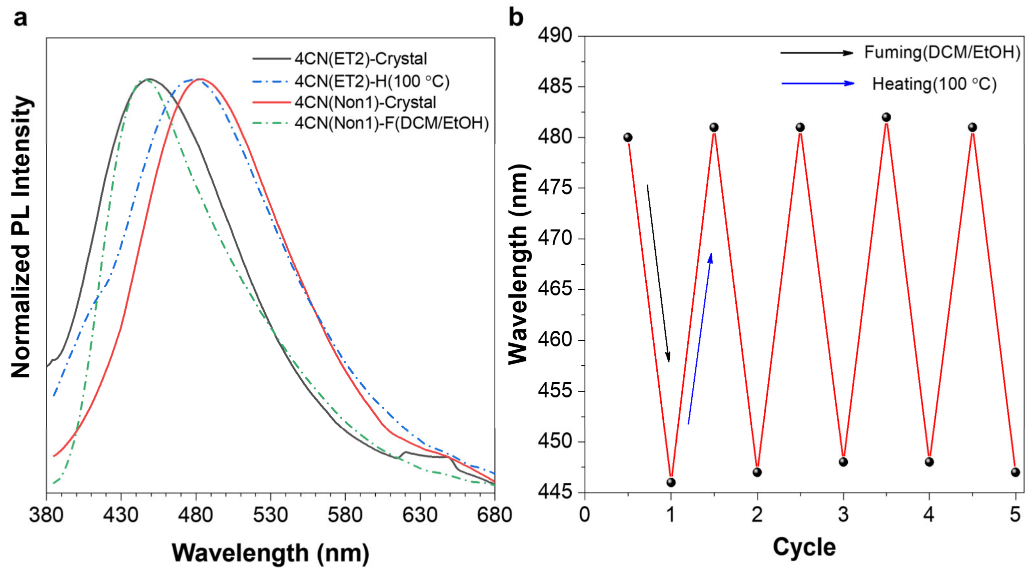

Supplementary Figure 39. a Normalized fluorescence spectra and **b** fatigue resistance of reversible conversion between 4CN(ET2) (**G1**) and 4CN(Non1) (**G3**).

Conversion from 4CN(Hex) (G2) to 4CN(Non1) (G3)

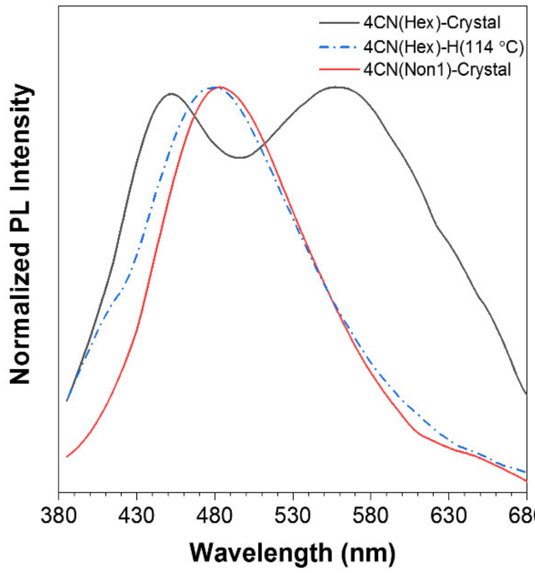

Supplementary Figure 40. Normalized fluorescence spectra of conversion from 4CN(Hex) (**G2**) to 4CN(Non1) (**G3**).

Reversible conversion between 4CN(THF) (G4) and 4CN(Non1) (G3)

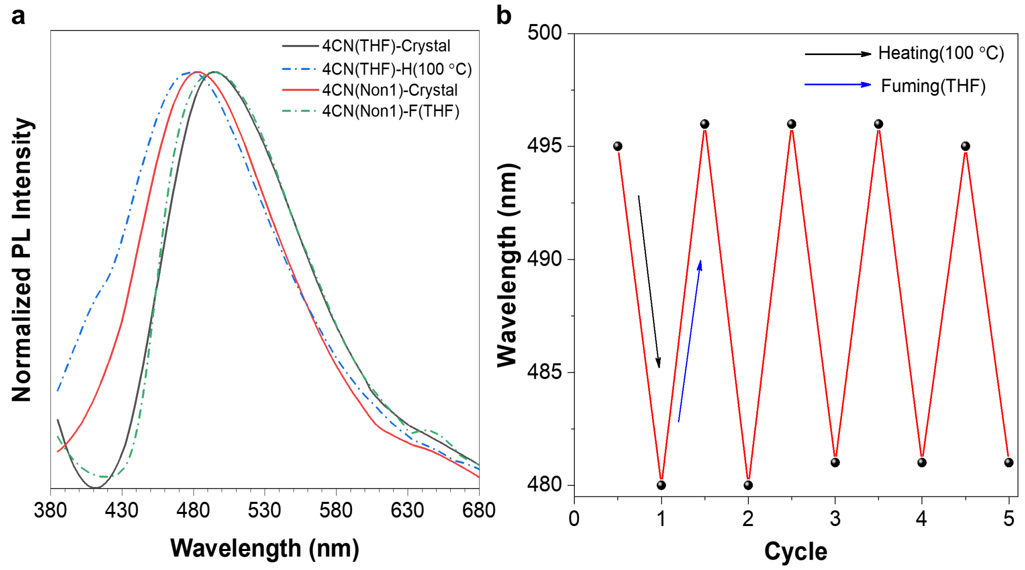

Supplementary Figure 41. a Normalized fluorescence spectra and **b** fatigue resistance of reversible conversion between 4CN(THF) (**G4**) and 4CN(Non1) (**G3**).

Reversible conversion between 4CN(THF) (G4) and 4CN(Hex) (G2)

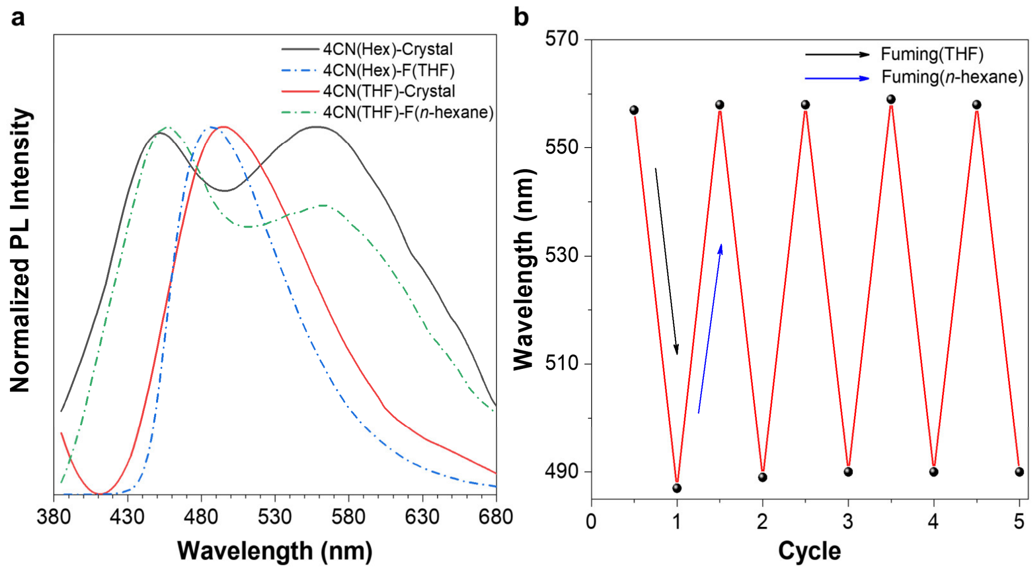

Supplementary Figure 42. a Normalized fluorescence spectra and **b** fatigue resistance of reversible conversion between 4CN(THF) (**G4**) and 4CN(Hex) (**G2**). For dual emission, the fatigue resistance was revealed by reversible maximum emission in the yellow emission region.

Reversible conversion between 4CN(THF) (G4) and 4CN(ET2) (G1)

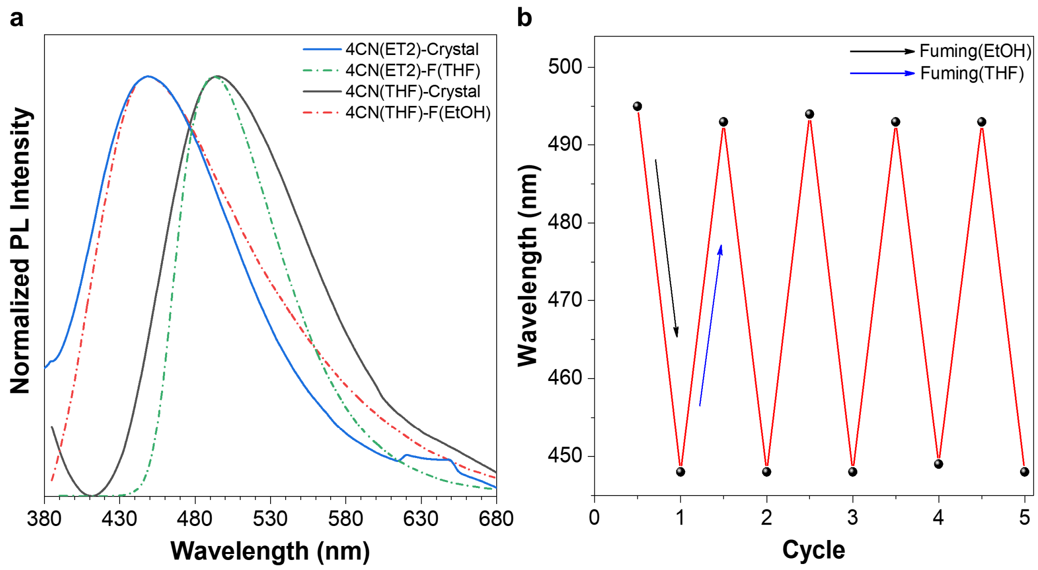

Supplementary Figure 43. a Normalized fluorescence spectra and **b** fatigue resistance of reversible conversion between 4CN(THF) (**G4**) and 4CN(ET2) (**G1**).

Conversion from 4CN(ET1) (G2) to 4CN(ET2) (G1) and conversion from 4CN(ET2) (G1) to 4CN(Hex) (G2)

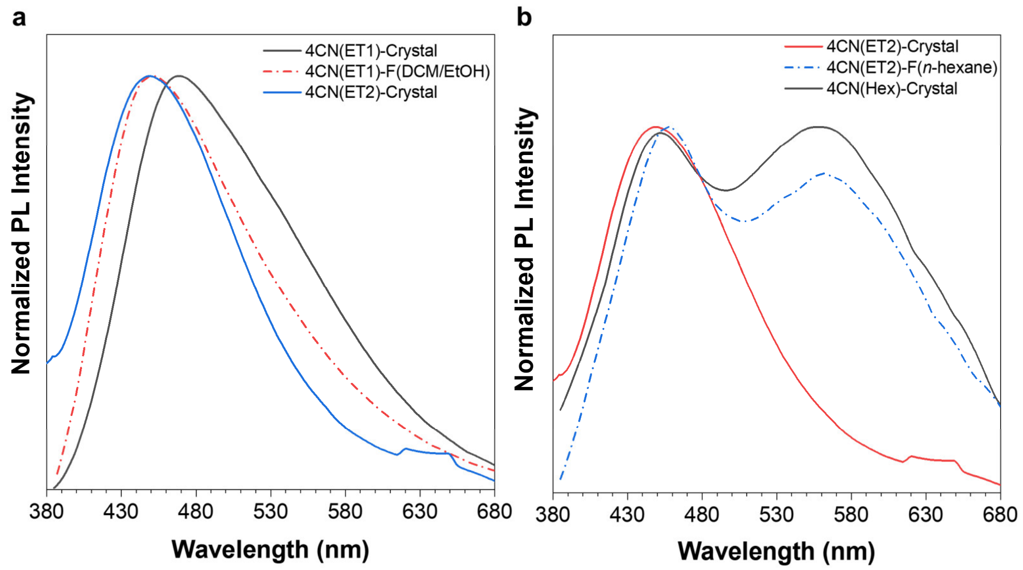

Supplementary Figure 44. Normalized fluorescence spectra of **a** conversion from 4CN(ET1) (**G2**) to 4CN(ET2) (**G1**) and **b** conversion from 4CN(ET2) (**G1**) to 4CN(Hex) (**G2**).

Supplementary Table 2. Crystal data for non-white-light emitting crystals

Entry	4CN(ET2)	4CN(MT)	4CN(Non2)	4CN(Non1)	4CN(DCM)	4CN(TCM)	4CN(THF)
Empirical formula	$(C_{30}H_{16}N_4)$ (C_2H_6O)	$(C_{30}H_{16}N_4)$ (CH_4O)	$C_{30}H_{16}N_4$	$C_{30}H_{16}N_4$	$(C_{30}H_{16}N_4)$ (CH_2Cl)	$(C_{30}H_{16}N_4)$ $(CHCl_3)$	$(C_{30}H_{16}N_4)$ (C_4H_4O)
Formula weight	478.53	460.48	432.47	432.47	517.39	551.83	500.54
Temperature/K	150	150	120	120	150	150	120
Crystal system	monoclinic	monoclinic	monoclinic	monoclinic	monoclinic	monoclinic	monoclinic
Space group	$P2_1/c$	$P2_1/c$	$P2_1/c$	C2/c	$P2_1/c$	$P2_1/c$	$P2_1/c$
a/Å	12.9854(6)	12.953	15.7974(8)	15.721	9.182	9.264	9.423
b/Å	15.0418(7)	14.863	9.7025(6)	9.835	12.862	12.832	12.902
c/Å	13.5120(6)	13.147	15.6259(8)	15.943	22.872	23.010	23.248
α/°	90	90	90	90	90	90	90
β/°	92.558(4)	90.15	98.297(4)	98.63	90.76	90.53	90.89
γ/°	90	90	90	90	90	90	90
Volume/$Å^3$	2636.6(2)	2531.0	2370.0(2)	2437.2	2701.2	2735.4	2825.9
Z	4	4	4	4	4	4	4
R_1/%	5.33	4.71	7.17	9.13	5.76	6.32	9.11
CCDC	2090909	2090907	2090905	2090904	2090910	2090903	2090906

Supplementary Table 3. Crystal data for white-light emitting crystals

Entry	4CN(Hex)	4CN(Hep)	4CN(Oct)	4CN(Dec)	4CN(Dod)
Empirical formula [a]	$(C_{30}H_{16}N_4)(C_6H_{14})_x$	$(C_{30}H_{16}N_4)(C_7H_{16})_x$	$(C_{30}H_{16}N_4)(C_8H_{18})_x$	$(C_{30}H_{16}N_4)(C_{10}H_{22})_x$	$(C_{30}H_{16}N_4)(C_{12}H_{26})_x$
Temperature/K	120	120	120	120	120
Crystal system	tetragonal	tetragonal	tetragonal	tetragonal	tetragonal
Space group	$I4_1/acd$	$I4_1/acd$	$I4_1/acd$	$I4_1/acd$	$I4_1/acd$
a/Å	22.2889(9)	22.1994(10)	22.2301(7)	22.2896(13)	22.2596(13)
b/Å	22.2889(9)	22.1994(10)	22.2301(7)	22.2896(13)	22.2596(13)
c/Å	20.7869(9)	21.0846(10)	20.9280(5)	20.7847(11)	20.9592(18)
α/°	90	90	90	90	90
β/°	90	90	90	90	90
γ/°	90	90	90	90	90
Volume/Å^3	10326.8(9)	10390.8(11)	10342.1(7)	10326.4(13)	10385.1(15)
Z	16	16	16	16	16
R_1/%	6.47	7.83	9.89	6.81	9.79
CCDC	2090908	2090911	2090902	2111294	2102622

[a]x: As the solvents in the channel are highly disordered, it is difficult to determine the exact ratio of solvent molecules to the framework.

Supplementary References

1. Bhunia, A., Vasylyeva, V. & Janiak, C. From a supramolecular tetranitrile to a porous covalent triazine-based framework with high gas uptake capacities. *Chem. Commun.* **49**, 3961-3963 (2013).
